# Drug Repurposing to Inhibit Oncostatin M in Crohn’s Disease

**DOI:** 10.3390/molecules30091897

**Published:** 2025-04-24

**Authors:** Faranak Bahramimehr, Axel Guthart, Stefanie Kurz, Yuanping Hai, Mona Dawood, Rümeysa Yücer, Nasim Shahhamzehei, Ralf Weiskirchen, Wilfried Roth, Wolfgang Stremmel, Gerhard Bringmann, Thomas Efferth

**Affiliations:** 1 Department of Pharmaceutical Biology, Institute of Pharmaceutical and Biomedical Sciences, Johannes Gutenberg University, Staudinger Weg 5, 55128 Mainz, Germany; fabahram@students.uni-mainz.de (F.B.); aguthart@students.uni-mainz.de (A.G.); stefanie_kurz@gmx.de (S.K.); haiyuanping2018@163.com (Y.H.); modawood@uni-mainz.de (M.D.); ryuecer@students.uni-mainz.de (R.Y.); nshahham@uni-mainz.de (N.S.); 2 Department of Molecular Biology, Faculty of Medical Laboratory Science, Al-Neelain University, Khartoum 11121, Sudan; 3 Institute of Molecular Pathobiochemistry, Experimental Gene Therapy and Clinical Chemistry (IFMPEGKC), RWTH University Hospital Aachen, 52074 Aachen, Germany; rweiskirchen@ukaachen.de; 4 Institute of Pathology, University Medical Center, Johannes Gutenberg University, 55131 Mainz, Germany; wilfried.roth@unimedizin-mainz.de; 5 Clinic for Internal Medicine, Beethovenstraße 2, 76530 Baden-Baden, Germany; wolfgangstremmel@aol.com; 6 Institute of Organic Chemistry, University of Würzburg, Am Hubland, 97074 Würzburg, Germany; gerhard.bringmann@uni-wuerzburg.de

**Keywords:** chronic inflammation, inflammatory bowel disease, microscale thermophoresis, molecular docking, transcriptomics, virtual drug screening

## Abstract

Crohn’s disease is an inflammatory bowel disease (IBD) that currently lacks satisfactory treatment options. Therefore, new targets for new drugs are urgently needed to combat this disease. In the present study, we investigated the transcriptomics-based mRNA expression of intestinal biopsies from patients with Crohn’s disease. We compared the mRNA expression profiles of the ileum and colon of patients with those of healthy individuals. A total of 72 genes in the ileum and 33 genes in the colon were differentially regulated. Among these, six genes were overexpressed in both tissues, including *IL1B*, *TCL1A*, *HCAR3*, *IGHG1*, *S100AB*, and *OSM*. We further focused on *OSM*/oncostatin M. To confirm the responsiveness of intestinal tissues from patients with Crohn’s disease to oncostatin M inhibition, we examined the expression of the oncostatin M using immunohistochemistry in patient biopsies as well as in kindlin-1^−/−^ and kindlin-2^−/−^ knockout mice, which exhibit an inflammatory bowel disease (IBD) phenotype, and found strong oncostatin M expression in all samples examined. Next, we conducted a drug-repurposing study using the supercomputer MOGON and bioinformatic methods. A total of 13 candidate compounds out of 1577 FDA-approved drugs were identified by PyRx-based virtual drug screening and AutoDock-based molecular docking. Their lowest binding energies (LBEs) ranged from −10.46 (±0.08) to −8.77 (±0.08) kcal/mol, and their predicted inhibition constants (pK_i_) ranged from 21.62 (±2.97) to 373.78 (±36.78) nM. Ecamsule has an interesting stereostructure with two C_2_-symmetric enantiomers (1*S*,4*R*-1′*S*,4′*R* and 1*R*,4*S*-1′*R*,4′*S*) (**1a** and **1b**) and one *meso* diastereomer (1*S*,4*R*-1′*R*,4′*S*) (**1c**). These three stereoisomers showed strong, albeit differing, binding affinities in molecular docking. As examined by nuclear magnetic resonance and polarimetry, the 1*S*,4*R*-1′*S*,4′*R* isomer was the stereoisomer present in our commercially available preparations used for microscale thermophoresis. Ecamsule (**1a**) was chosen for in vitro validation using recombinant oncostatin M and microscale thermophoresis. Considerable dissociation constants were obtained for ecamsule after three repetitions with a K_d_ value of 11.36 ± 2.83 µM. Subsequently, we evaluated, by qRT-PCR, the efficacy of ecamsule (**1a**) as a potential drug that could prevent oncostatin M activation by inhibiting downstream inflammatory marker genes (*IL6*, *TNFA*, and *CXCL11*). In conclusion, we have identified oncostatin M as a promising new drug target for Crohn’s disease through transcriptomics and ecamsule as a potential new drug candidate for Crohn’s disease through a drug-repurposing approach both in silico and in vitro.

## 1. Introduction

For many decades, the treatment of inflammatory bowel disease (IBD) represented a major challenge among all chronic inflammatory diseases [1,2]. The complex and still poorly understood pathophysiology is a main factor causing the severity of IBD, ranging from disturbances in immune responses, genetic predisposing factors, and viral infections to changes in the intestinal microbiota [3]. Despite the availability of treatments for IBD, its incidence is increasing [4,5] and it is spreading in many regions of the world, including Asia, Africa, Europe, and the United States. It was estimated that more than 290 million people were affected by this disease in November 2022 [6]. The increasing urbanization in many parts of the world in the 21st century has further accelerated the incidence of IBD [7]. Even worse, the well-known predisposition for cancer due to chronic inflammation also applies to IBD. Patients with IBD have a two- to three-fold higher risk of developing colitis-associated colorectal cancer [8].

Morbus Crohn or Crohn’s disease and ulcerative colitis are the two most common types of IBD [9,10]. Crohn’s disease is often described as a complex mucosal barrier disease, where bacteria are not effectively fought due to a discontinuing manifestation pattern with leaky intestinal epithelia in the ileum and colon. Immune system-inhibiting drugs are used for therapy due to the involvement of immune reactions in the disease process. Glucocorticoids, mostly recommended as short-term therapy, and amino salicylates are typically the first choices for drug treatment [11]. Azathioprine, methotrexate, and cyclosporin A (as a rescue therapeutic strategy) are commonly used immunosuppressive drugs that reduce pro-inflammatory cytokine secretion, leading to an increased anti-inflammatory response [12,13]. However, these medications are older and have been used for a long time, with many patients not responding adequately to them or experiencing adverse reactions. Therefore, therapeutic monoclonal antibodies have been developed [14]. Established antibodies that target tumor necrosis factor α (TNF-α) as a contributing factor to Crohn’s disease include infliximab, adalimumab, and other monoclonal antibodies [15]. AVX-470 is a previously developed polyclonal bovine-derived antibody against TNF-α [16]. Other medications include antibodies against integrins (e.g., natalizumab, ontamalimab, etrolizumab, and abrilumab), small molecules targeting Janus kinase, and other drug candidates in clinical trials [17]. With the increasing number of patients with IBD, especially Crohn’s disease, and the high percentages of non-responding cases (20–34%) [18], there is an urgent need to discover novel druggable targets to combat Crohn’s disease.

In this context, the question arises of how potential targets for the development of drugs against Crohn’s disease can be identified. Well-known targets include pro-inflammatory cytokines such as TNF-α, IL-1β, and others. The advent of so-called “-omics” technologies tremendously facilitated the identification of novel potential drug targets for many different diseases [19,20]. The interactome of genomics, transcriptomics, proteomics, and metabolomics has also been discussed as a resource for target identification in IBD and Crohn’s disease [9].

In the present investigation, we first identified oncostatin M as a novel target for the treatment of Crohn’s disease. This was achieved using transcriptome-wide mRNA expression profiling and immunohistochemistry, which revealed that this protein is overexpressed in intestinal biopsies. Oncostatin M belongs to the IL-6 cytokine family and has a complex receptor composed of the oncostatin M receptor (*OSMR*) (type II) or the leukemia inhibitory factor receptor-β (*LIFR*) (type 1) and gp130 co-receptor. Depending on the cell type and environmental conditions, each of these receptors can trigger various signaling pathways, leading to the immune response to Crohn’s disease [21].

Next, we applied a drug-repurposing approach and performed a supercomputer-based virtual drug screening with 1577 FDA-approved drugs. The drug screening results were validated by molecular docking in silico and microscale thermophoresis in vitro. Furthermore, we employed differentiated CaCo-2 cells and stimulated them with OSM to recapitulate, in vitro, the link of OSM to inflammation by biochemical assays. Then, we applied ecamsule at varying concentrations to CaCo-2 cells to determine whether this compound inhibits the effect of OSM and verified the OSM-inhibitory effect of ecamsule by qPCR and monitoring inflammatory key genes. Finally, we highlight that ecamsule can occur in three possible stereoisomeric forms—a fact that has not been sufficiently recognized so far, even though the drug has been marketed for many years.

## 2. Results

### 2.1. Transcriptomics of Clinical Colon Biopsies of Patients with Crohn’s Disease

**Figure 1:**
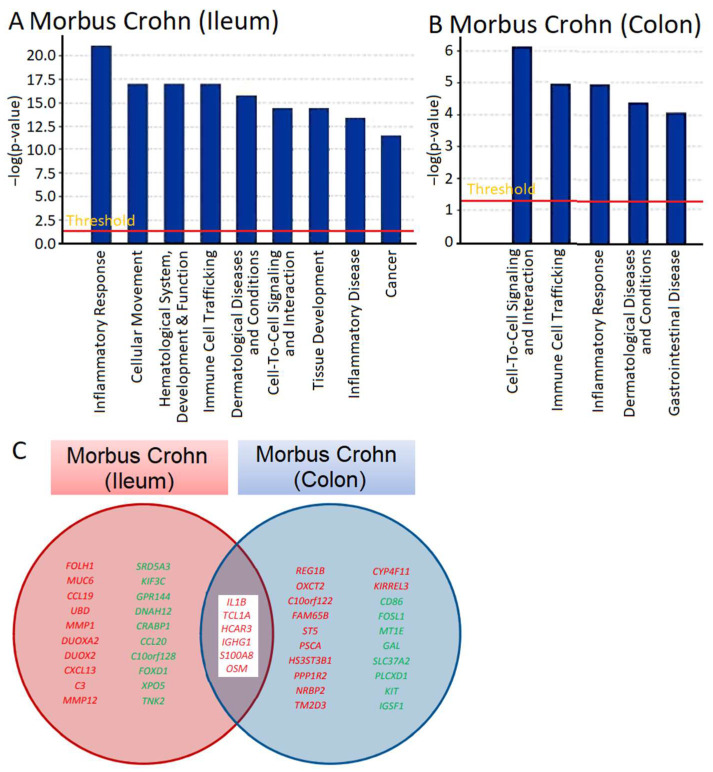
Transcriptomic analysis and gene expression profiling of intestinal biopsies from patients suffering from Crohn’s disease. The gene expression in the ileum and colon biopsies was compared with that in ileum and colon samples from healthy individuals, and the differentially expressed genes were identified. Ingenuity Pathway Analysis software (content version: 51963813, Release Date: 11 March 2020) (IPA; Ingenuity Systems, Redwood City, CA, USA) was employed for visualization. (**A**,**B**) show cellular functions and disease pathways in the ileum and colon samples. The Venn diagram in (**C**) depicts genes that were overexpressed in both the ileum and colon biopsies of patients with Crohn’s disease, with OSM presented alongside these genes.

mRNA expression across the transcriptome was measured using microarray hybridization and analyzed with Chipster software (version 3.16.3) [22]. A total of 72 genes were found to be differentially expressed, with 62 upregulated and 10 downregulated, in ileum samples, and 33 genes, 25 upregulated and 6 downregulated, in colon biopsies of patients with Crohn’s disease compared to samples from healthy individuals (Tables S1 and S2). We utilized Ingenuity Pathway Analysis (IPA) software (content version: 51963813, Release Date: 11 March 2020) (IPA; Ingenuity Systems, Redwood City, CA, USA) to determine which cellular functions and disease pathways were impacted by Crohn’s disease. The results for ileum samples are presented in Figure 1A and those for colon biopsies in Figure 1B. We observed various cellular functions related to inflammation and immunology, such as inflammatory response and immune cell trafficking in both ileum and colon biopsies and inflammatory disease in colon biopsies. This aligns with what is expected for IBDs like Crohn’s disease, validating the plausibility of transcriptomic analysis. Other categories identified by IPA were also associated with immunological processes, including cellular movement, hematological alterations, and the development and function of immune response, as well as cell-to-cell signaling and interaction. The presence of gene clusters related to dermatological diseases and immune disorders, cancer, and gastrointestinal diseases is significant for understanding immunological processes that may be relevant for the manifestation of Crohn’s disease.

We searched not only for genes that were differentially expressed in comparison to normal colon tissue, but also for genes that were deregulated in both the ileum and colon of patients with Crohn’s disease (Tables S1 and S2). As shown in Figure 1C, some of the genes that were overexpressed in both the ileum and colon included *IL1B*, *TCL1A*, *HCAR3*, *IGHG1*, *S100AB*, and *OSM.* These genes are all involved in inflammatory processes and immune responses to some extent. Of particular interest to us was *OSM*, as it encodes a cytokine, oncostatin M, that regulates the production of other cytokines and may therefore be a highly relevant master key for the manifestation of inflammatory processes in Crohn’s disease.

**Figure 2:**
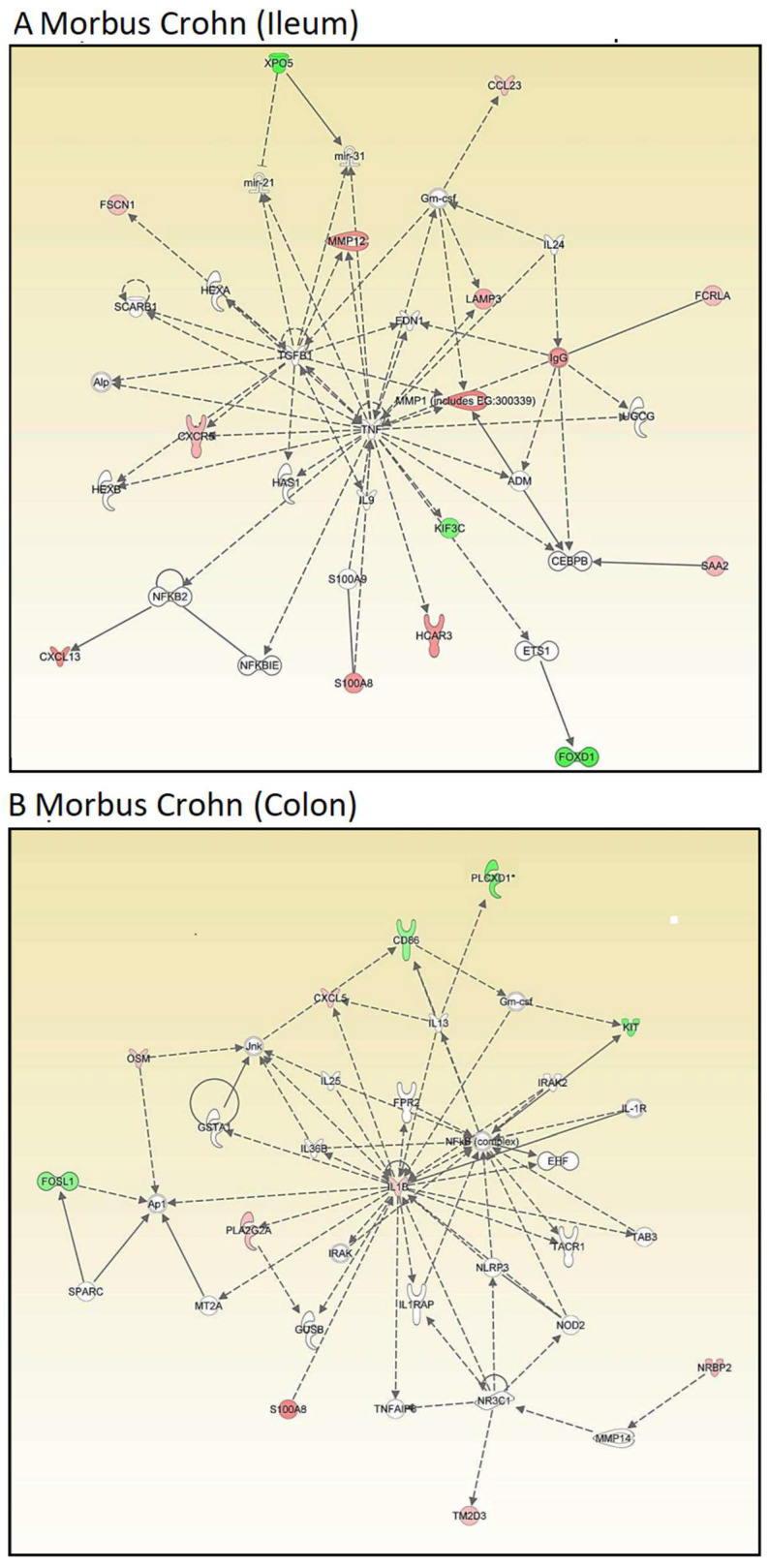
Transcriptomics-based unsupervised network analysis of gene expression profiles of intestinal biopsies from patients suffering from Crohn’s disease as determined by Ingenuity Pathway Analysis. (**A**) Ileum samples and (**B**) colon samples. Red color indicates overexpression, green color indicates downregulation.

Then, we conducted network analyses using IPA. Genes were organized regardless of their cellular functions and disease pathways to explore novel interactive relationships. IPA identifies networks of gene sets based on known interactions published in the literature. TNF and IGFB1 (ileum, Figure 2A) and IL1B and NFKB (colon, Figure 2B) emerged as central nodes in the interaction networks for the ileum and colon, suggesting potential roles in the regulation of signal transduction. However, many network genes appeared with white symbols, indicating their unresolved functional significance in this specific context. The networks are created by IPA using the upregulated genes (shown by red symbols) and downregulated genes (shown by green symbols) to form a network. To complete the signaling network, IPA uses white symbols to represent missing elements (i.e., genes that are neither up- nor downregulated). Figure 2A,B show numerous white elements, indicating that these networks may have limited explanatory power for molecular signaling in Crohn’s disease.

**Figure 3:**
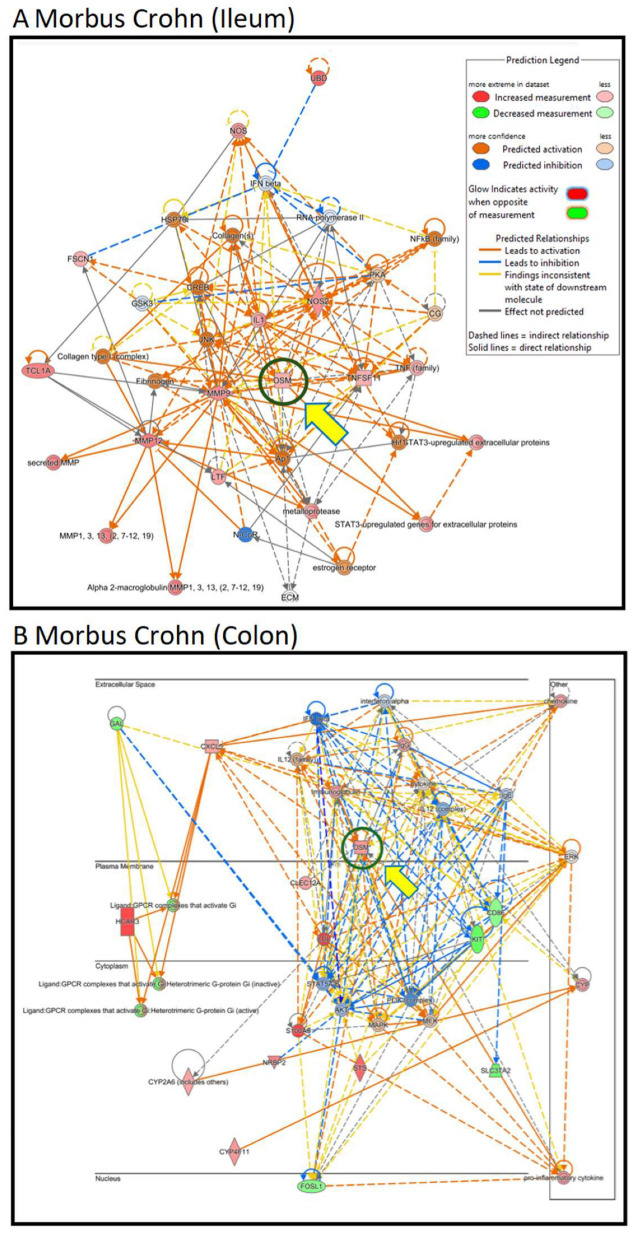
Transcriptomics-based supervised network analysis with OSM as a central player in the gene interaction networks of intestinal biopsies from patients suffering from Crohn’s disease as determined by Ingenuity Pathway Analysis. (**A**) Ileum samples and (**B**) colon samples. Color and line settings are explained in the insert of (**A**).

Therefore, we focused on OSM. Even though oncostatin M did not appear as a central node in the kinds of unsupervised interaction networks shown above, we speculated that it may play an important role in Crohn’s disease. As a result, we generated supervised interaction networks using IPA with OSM as a central element for network construction. The interaction networks in Figure 3A (for ileum) and Figure 3B (for colon) show that OSM, as the central node of the networks, interacted with other genes that were all overexpressed (illustrated by red symbols). Therefore, we concluded that the OSM signaling networks were more significant than the previous networks and that OSM may be a relevant target for drug development to combat Crohn’s disease.

### 2.2. Immunohistochemistry of Clinical Biopsies of Patients with Inflammatory Bowel Diseases and Kindlin-1/2 Knockout Mice

**Figure 4:**
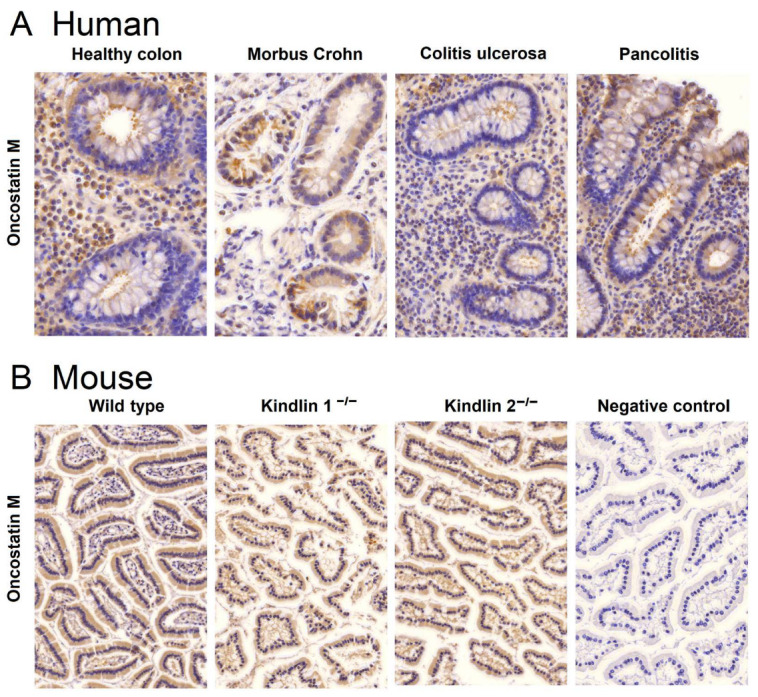
Immunohistochemical detection of oncostatin M in formalin-fixed paraffin-embedded colon tissues of (**A**) healthy individuals and patients suffering from Crohn’s disease, ulcerative colitis, and the ulcerative colitis subgroup of pancolitis and (**B**) wild-type mice and kindlin-1 and kindlin-2 knockout mice with their respective ulcerative colitis phenotype. The negative control was without a primary antibody. Magnification: (**A**) 64×; (**B**) 20×.

We performed immunohistochemical staining of human colon tissues obtained from healthy individuals and patients suffering from Crohn’s disease and ulcerative colitis, including the ulcerative colitis subgroup of pancolitis, to study whether oncostatin M protein is expressed in colonic tissues. Since the oncostatin M can be found in various tissues and cells, such as brain cells and osteoblasts, we aimed to confirm if the high oncostatin M gene expression observed in transcriptomic profiling corresponds to high OSM expression in biopsies. Figure 4A illustrates that OSM was present in all examined colonic tissues, albeit with varying histological structures and intensities. Cytoplasmic and nuclear expressions were detected in lamina propria cells. Crypt cells displayed noticeable membrane-bound staining, particularly at the lumen, facing the apical regions of the crypts. In Crohn’s disease tissues, expression was not only membrane-bound but also localized in the cytoplasm of crypt cells exhibiting stronger intensity compared to other tissues.

In addition to human tissues, we also examined murine colon biopsies. Biopsies from wild-type, kindlin-1^−/−^, and kindlin-2^−/−^ mice were used for this study. Tissues from knockout mice were chosen, because gene silencing in these genes disrupts the mucus barrier and causes immunomodulation similar to chronic IBD in humans [23,24]. Immunohistochemical analyses showed comparable OSM expression in colon tissues from wild-type and knockout mice (Figure 4B). The specificity of the immunohistochemical reactions was confirmed by omitting the primary antibody during the staining procedure. A representative example of such a negative control is depicted in Figure 4B on the right side.

### 2.3. Virtual Drug Screening with PyRx and Molecular Docking with AutoDock for Oncostatin M Inhibitors

**Figure 5:**
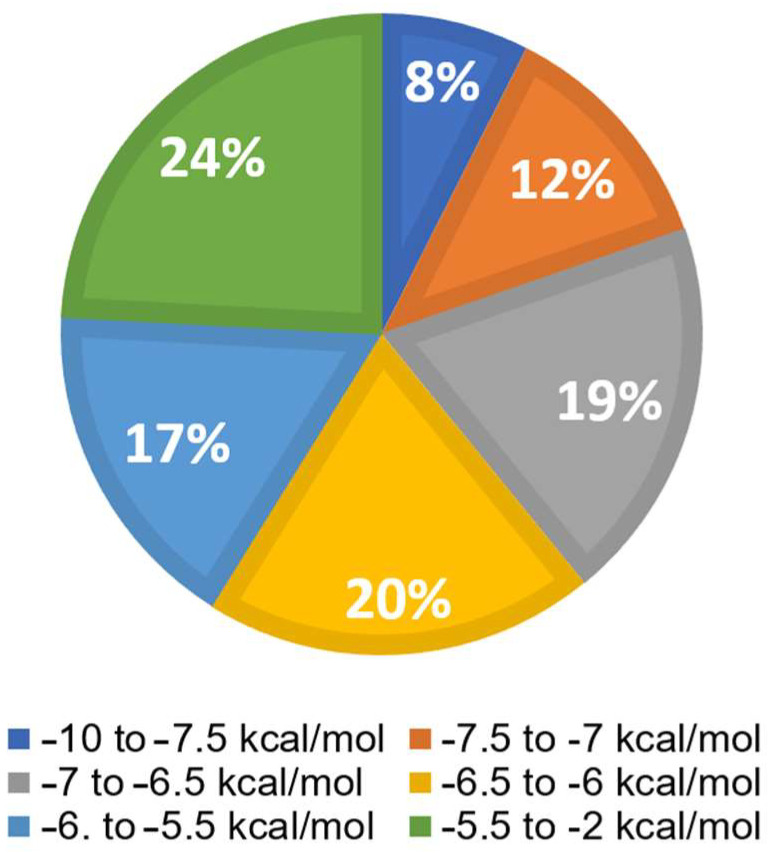
Virtual drug screening and molecular docking of 1577 FDA-approved drugs for their interaction with oncostatin M using PyRx. The distribution of lowest binding energy (LBE) values is shown in six groups, from −10 to −2 kcal/mol.

Virtual screening was performed using Python Prescription PyRx (Version 0.8). Available online: https://pyrx.sourceforge.io/ (accessed on 31 March 2023) with a subset of the ZINC database, which included 1577 FDA-approved compounds. The bar diagram in Figure 5 displays six groups. The top 8% of the 1577 substances exhibited the lowest binding energies (LBEs) within a range of −10 to −7.5 kcal/mol, making them potential candidates for oncostatin M inhibition. Following this, 12% of the compounds had LBE values falling between −7.5 and −7 kcal/mol. The remaining groups demonstrated weak binding affinities (>−7 kcal/mol). The lowest binding energy (LBE) represents a parameter of the strength of the interaction between a test compound and its target protein. Low LBE values indicate strong and stable interactions, which means that the binding affinity between two partners is high.

In the next step, the top 50 molecules resulting from the screening were selected for molecular docking using AutoDock 1.5.6, based on their lowest binding energy with the OSM protein of PyRx. To ensure the quality of the molecular docking calculations, we correlated the lowest binding energies (LBEs, kcal/mol) with the predicted inhibition constants (pKi, nM). As expected, both parameters showed a significant correlation with each other among this set of selected compounds (*r* = 0.998; *p* < 0.0001; Spearman rank correlation test).

**Figure 6:**
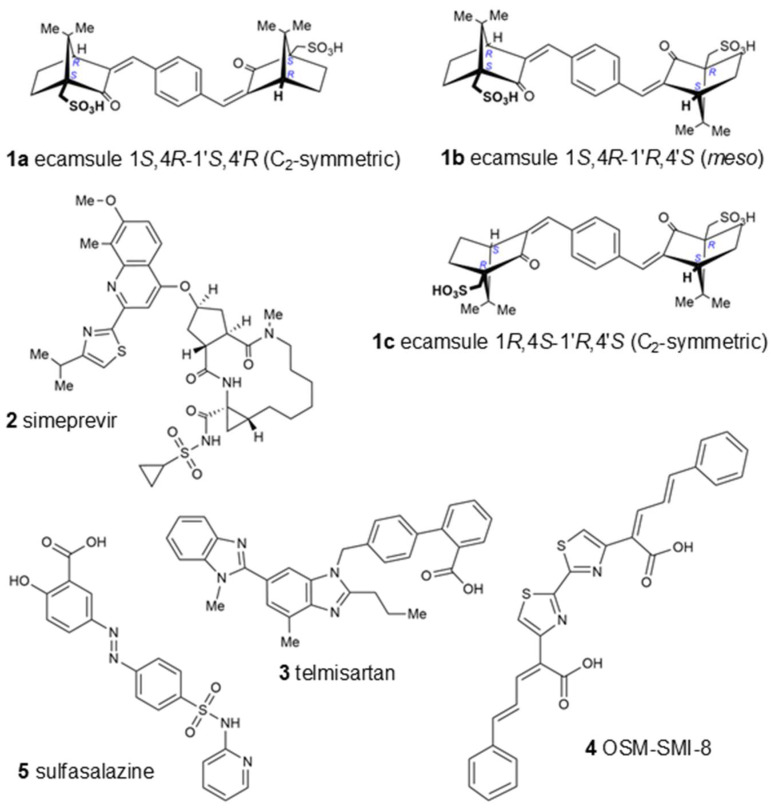

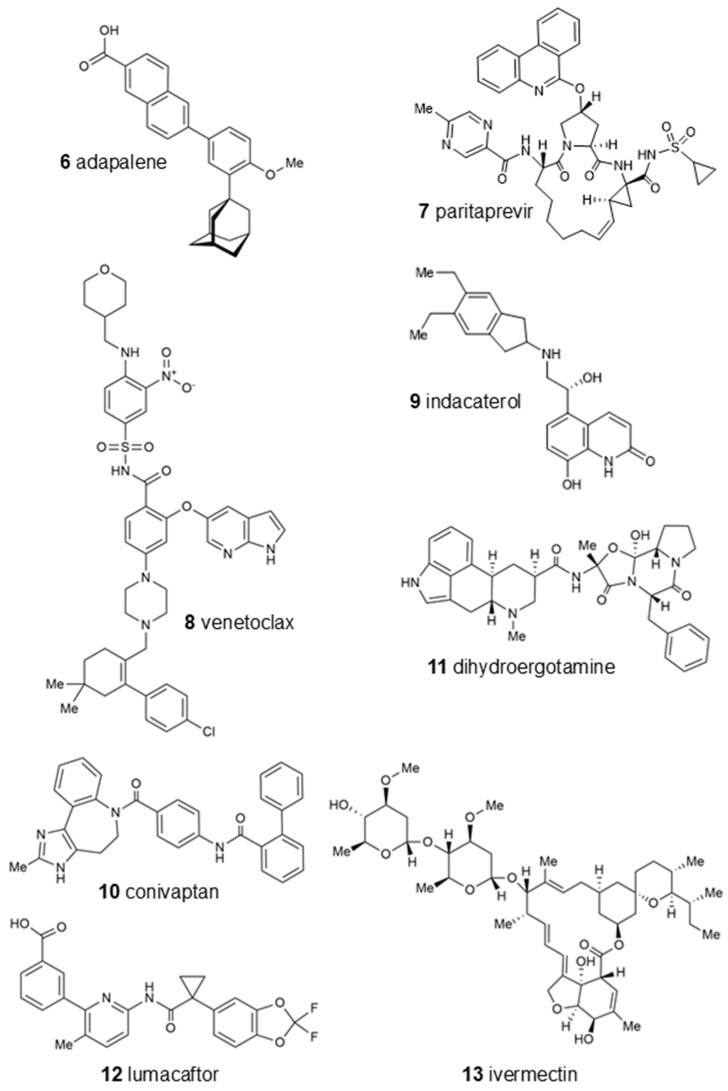
Chemical structures of top-ranked FDA-approved drugs binding in silico to oncostatin M. OSM-SMI-8 served as a control drug known to inhibit oncostatin M. Ecamsule appears as three stereoisomers: two enantiomers, compounds **1a** and **1c**, and a *meso* form, **1b**.

Table 1 displays the LBE values and the predicted inhibition constants (pK_i_) of the 13 candidates binding to oncostatin M, which were then used in further tests. Among them were drugs from a variety of pharmacological classes, such as drugs against viruses (paritaprevir), nematodes (ivermectin), hypertension (telmisartan), cancer (venetoclax, OSM-SMI8 control drug), migraine and dementia (dihydroergotamine), low sodium levels (conivaptan), or cystic fibrosis (lumacaftor). Furthermore, dermatics (ecamsule, adapalene), the immunomodulator sulfasalazine, and the bronchodilator indacaterol appeared as oncostatin M-binding compounds. This wide diversity indicates that specific features of their chemical structures themselves, rather than their pharmacological applications, qualified these compounds as oncostatin M ligands in our drug screening approach (Figure 6).

Interestingly, the UVA-light-filtering sunscreen compound ecamsule (**1**) also appeared among these top-ranked compounds. Upon an inspection of the chemical structure of ecamsule, it is evident that this substance, with its symmetric constitution and identical relative configurations within the two molecular halves, can exist in three different stereochemical forms: two C_2_-symmetric isomers and a *meso* form. Ecamsule possesses a symmetric constitution, synthetically arising from the double aldol condensation of *para*-terephthalaldehyde with two molecules of camphor sulfonic acid. This chiral precursor can be 1*S*,4*R*- or 1*R*,4*S*-configured—or racemic—so that ecamsule (**1**) can occur in three possible stereoisomeric forms, represented by structures **1a** (1*S*,4*R*-1′*S*,4′*R*), **1b** (1*S*,4*R*-1′*R*,4′*S*), and **1c** (1*R*,4*S*-1′*R*,4′*S*). Among these, **1a** and **1c**, with their identical molecular halves, are chiral, C_2_-symmetric, and enantiomeric to each other, while **1b**, with its enantiomorphic (mirror-imaged) molecular halves, is a *meso* compound, with an inner mirror plane, and is, thus, achiral and diastereomeric to both **1a** and **1c**.

The high binding energies of ecamsule made it worthwhile to analyze these three possible stereoisomers separately, using PyRx and AutoDock techniques to explore the influence of stereostructure on their pharmacological properties. Figure 6 shows the three different stereostructures of ecamsule, compounds **1a**, **1b**, and **1c**. The fact that these three stereoisomers showed significantly different binding properties proves the relevance of the—often neglected—stereochemical aspect. The specific diversification of the binding properties can be seen from the fact that compound **1a** is the top-ranked agent, while **1b** and **1c** are ranked slightly weaker, below simeprevir (**2**), further proving the high stereochemical relevance. Thus, the structure of ecamsule as its 1*S*,4*R*-1′*S*,4′*R* (C_2_-symmetric) isomer, i.e., compound **1a**, exhibited the lowest binding energy compared to the other stereoisomers—and all other investigated structures—with **1a** ranking as the strongest-binding compound out of the 13 compounds analyzed in the AutoDock analysis.

**Figure 7:**
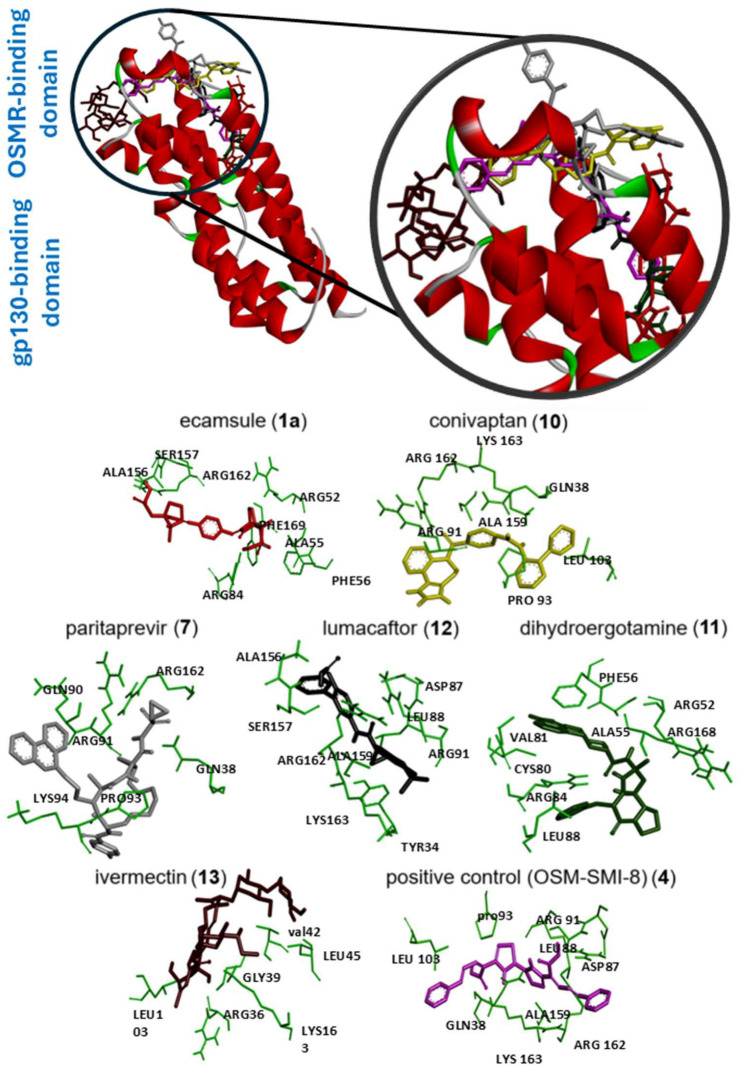
Molecular docking of six selected drugs binding to oncostatin M using AutoDock. Shown are 3D representations of the drugs and amino acids. Green color indicates the amino acid residues of oncostatin M; the other colors show the different ligands.

**Figure 8:**
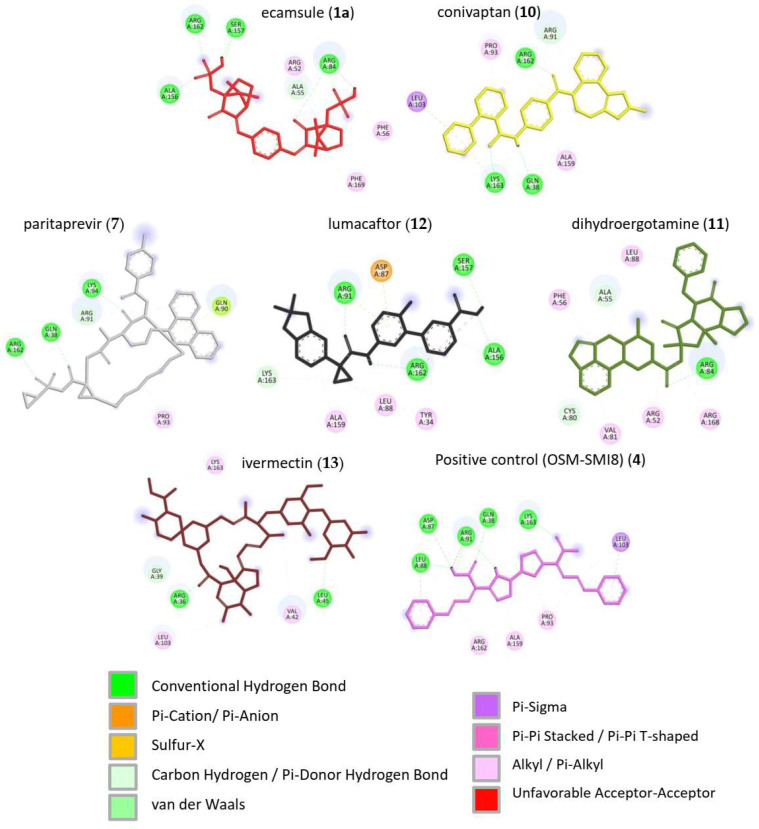
Molecular docking of six selected drugs binding to oncostatin M using AutoDock. Shown are 3D representations of the drugs and binding types to the amino acids.

Next, we visualized binding using VMD representations to inspect whether our candidates interact with oncostatin M at the same binding site as the control drug, OSM-SMI8. The interaction in 2D and 3D representations is shown in Figure 7 and Figure 8. In Figure 7, it is clear that the selected compounds bound to the same domain as OSM-SMI-8, specifically the binding site of the oncostatin M receptor, rather than the gp130 co-receptor. The bound amino acids were, however, partially different. The compounds investigated preferentially bound by forming hydrogen bonds, π-σ, and alkyl/π-alkyl bonds (Figure 8). The number of interacting amino acids was nine for OSM-SMI-8 and ranged from six to nine for all other drugs. The control drug OSM-SMI-8 shared the following amino acid residues with test compounds: GLN38 (conivaptan, paritaprevir), AAP87 (lumacaftor), LEU88 (lumacaftor, dihydroergotamine), ARG91 (conivaptan, paritaprevir, lumacaftor), PRO93 (paritaprevir), LEU103 (conivaptan, ivermectin), LYS163 (lumacaftor), and ALA193 (conivaptan, lumacaftor).

**Figure 9:**
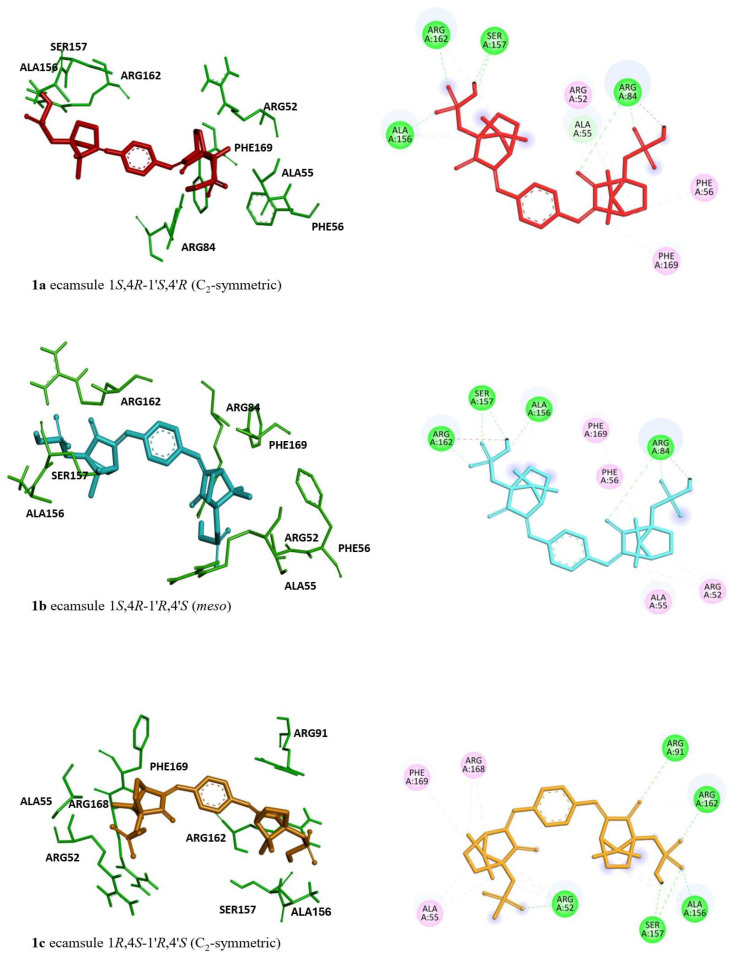
Molecular docking of the ecamsule stereoisomers (**1a**, **1b**, and **1c**) to oncostatin M by AutoDock. Structures **1a** and **1c** are enantiomeric to each other, while structure **1b** is a diastereomer. Shown are 3D representations of **1a**, **1b**, and **1c** with the interacting amino acids (left side) and 2D representations of their binding types to the amino acids (right side).

Regarding the stereoisomers of ecamsule, we found that all three forms interacted with eight amino acids, and four of these were shared by all three stereoisomers, including ALA55, PHE169, ALA156, and ARG162 (Figure 9).

### 2.4. Molecular Docking to Human Oncostatin M Using MOE

**Figure 10:**
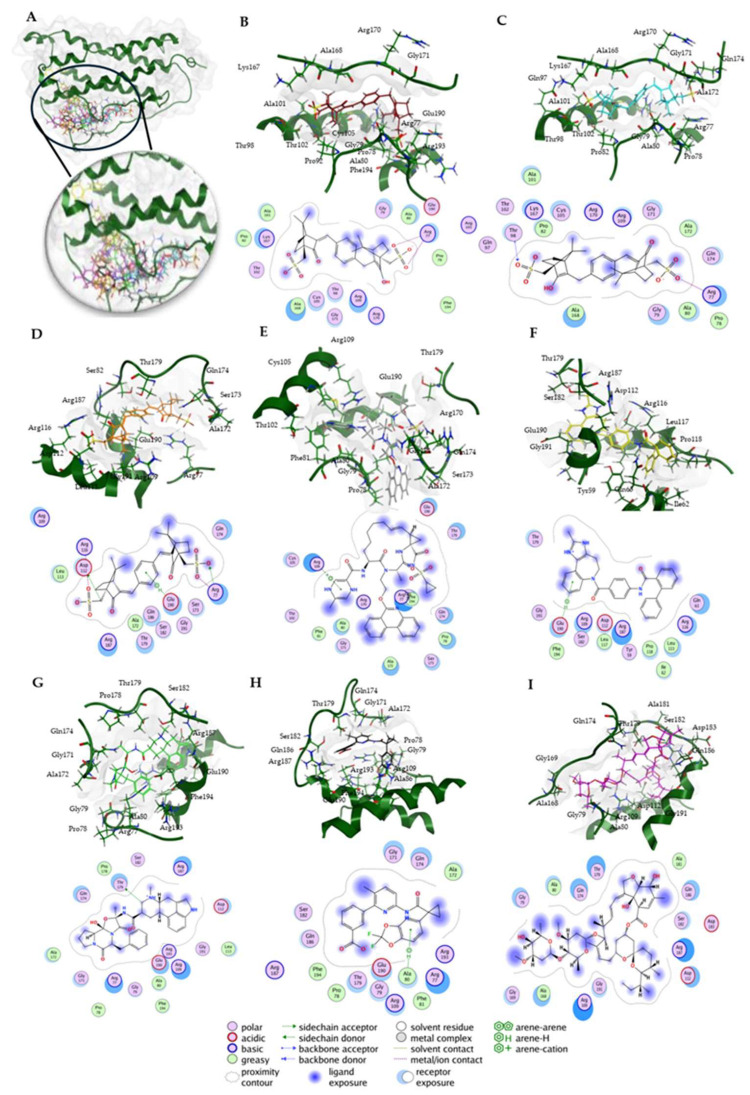
Molecular docking of human oncostatin M (PDB ID: 8V29) with candidate inhibitors using MOE 2022.02. (**A**) The complete structure of oncostatin M (dark green) is shown, along with the best docking poses of all eight compounds. (**B**–**I**) The individual binding poses of the eight compounds are displayed, each including a 3D view within the binding site and a corresponding 2D interaction map. The interaction cutoff was set at 4.5 Å. The order of compounds from B to I is as follows: **1a** (ecamsule, 1*S*,4*R*-1′*S*,4′*R* isomer, C_2_-symmetric) (red), **1b** (ecamsule, 1*S*,4*R*-1′*R*,4′*S* isomer, *meso*) (cyan), **1c** (ecamsule, 1*R*,4*S*-1′*R*,4′*S* isomer, C_2_-symmetric) (orange), **7** (paritaprevir) (gray), **10** (conivaptan) (yellow), **11** (dihydroergotamine) (green), **12** (lumacaftor) (black), and **13** (ivermectin) (magenta).

Then, a docking study with Molecular Operating Environment (MOE) software version 2022.02 from the Chemical Computing Group (https://www.chemcomp.com, accessed on 8 December 2024) was conducted to further validate the molecular docking results obtained from AutoDock. Eight candidate compounds were docked to human oncostatin M (Figure 10); their predicted binding affinities (docking score S) ranged from strong to less strong binders (Table 2). Notably, the macrocyclic drugs ivermectin (**13**) (−10.48 ± 0.32) and paritaprevir (**7**) (−9.87 ± 0.56) achieved the most favorable scores.

Among the smaller molecules, the ecamsule stereoisomers (compounds **1a**–**1c**) exhibited robust scores that ranged from −7.89 to −7.95, comparable to or better than those of several larger compounds. In contrast, the weakest binder was lumacaftor (**12**) (−7.41 ± 0.20 kcal/mol), which had the least favorable score in the set.

The pose of each compound was further evaluated by its Root Mean Square Deviation (RMSD) refine value. Most ligands exhibited a low RMSD refine value (approximately 1–2 Å), suggesting that the binding pose remained stable upon final refinement. For example, the RMSD refine values of ecamsule (**1a**) (1.65 ± 0.01) and conivaptan (**10**) (1.12 ± 0.01) indicate minor shifts during pose refinement. Such low values imply high confidence in the pose, as a top-ranked docking orientation within ~2 Å of an initial placement is generally considered a successful, well-converged result [25]. A few compounds showed higher RMSD refine values, notably paritaprevir (**7**) (3.21 ± 1.90 Å) and dihydroergotamine (**11**) (2.56 ± 0.00 Å), which suggests that these poses required larger adjustments or may occupy multiple orientations. In the context of molecular docking, an RMSD under ~2 Å is often used as a threshold for a reliably predicted binding mode [26]. The low RMSD refine values (with just one case surpassing 3 Å) validate the quality of the predicted complexes. In summary, the docking results suggest that the compounds exhibit potent in silico binding to OSM, and the refinement deviations were minor for the majority, reinforcing the stability of the predicted poses.

An analysis of ligand–OSM interactions identified Arg77, Arg109, and Glu190 as residues frequently interacting with the tested compounds (Table 2). Arg109, located in helix C, is part of the receptor-binding interface [27] and was present in all docking results. Arg77, situated in helix B, is involved in gp130 receptor recruitment [27] and appeared in 87.5% of docking poses. Glu190, positioned near helix D at the C-terminus, plays a role in electrostatic interactions crucial for receptor complex formation [27]. Ligand binding at these sites could sterically hinder receptor engagement or induce allosteric changes, potentially affecting cytokine signaling and receptor recognition.

To verify the specificity of ligand binding, molecular docking was performed against three negative control proteins: keratin (PDB ID: 6EC0, KRT1/KRT10 heterocomplex), ubiquitin (PDB ID: 8ST7), and protein kinase A (PKA, PDB ID: 1ATP). These controls were selected based on their structural and functional diversity to evaluate potential non-specific interactions. Keratin, a structural protein [28], was chosen to test for general surface binding, while ubiquitin, a small, globular protein [29], serves as a control for non-target-specific binding in cellular environments. PKA, an ATP-dependent kinase [30], was included to evaluate whether the compounds exhibit off-target binding to well-defined and conserved enzymatic binding pockets. To further investigate the specificity of the docking procedures, we performed docking with “dummy” proteins presumably not binding to our candidate compounds. Docking was performed with the same protocol as for OSM, with blind docking across the entire structure for keratin and ubiquitin, and defined docking at the ATP-binding site for PKA.

**Figure 11:**
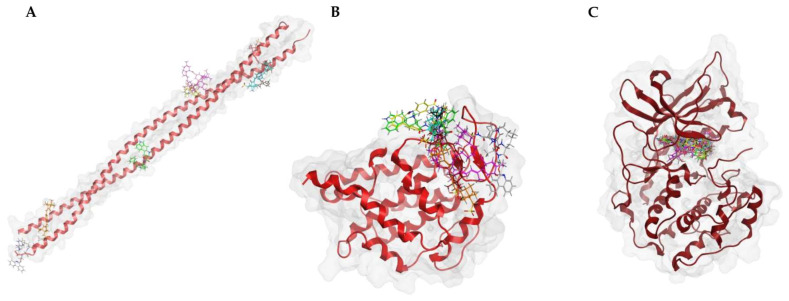
Docking poses of the top-scoring compounds for each negative control protein. (**A**) Keratin (PDB 6EC0), (**B**) ubiquitin (PDB 8ST7), and (**C**) protein kinase A (PKA, PDB 1ATP) were used as dummy proteins to assess non-specific binding. Blind docking was performed on keratin and ubiquitin, showing diverse binding locations, while PKA was docked at the ATP-binding pocket.

The results are summarized in Table 3. Docking scores for the negative controls were generally less favorable than for OSM, which indicates a lower binding affinity. However, PKA exhibited relatively strong binding for certain compounds, particularly conivaptan (**10**) (−8.79 ± 0.17). This suggests possible off-target interactions with kinase domains. By contrast, the ecamsule stereoisomers (**1a**–**1c**) displayed positive docking scores and, as a result, exhibited no binding to PKA, confirming their specificity for oncostatin M. Keratin and ubiquitin displayed weaker and more scattered binding patterns, which is expected for proteins without defined small-molecule-binding pockets (Figure 11).

**Figure 12:**
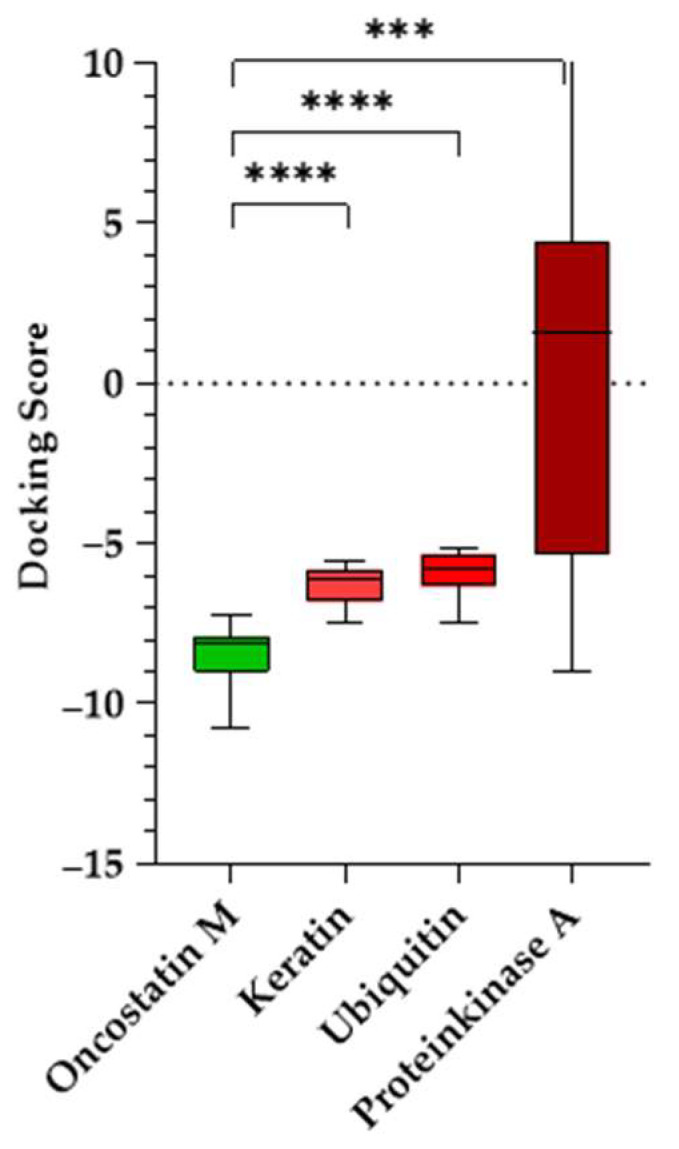
Docking scores of tested compounds against oncostatin M and negative control proteins (keratin, ubiquitin, and protein kinase A). Boxplots represent the distribution of docking scores, with the median, interquartile range, and whiskers indicating the minimum and maximum values. Statistical analysis was performed using Welch’s ANOVA followed by Games–Howell post hoc test (**** *p* < 0.0001; *** *p* < 0.001).

A Welch’s ANOVA test [31] was conducted to determine whether docking scores significantly differed between OSM and the negative controls, followed by Games–Howell post hoc tests [32] (Figure 12). The analysis confirmed a highly significant difference in docking scores, S, across the four proteins. OSM docking scores were significantly lower than those for the control proteins: keratin (*p* < 0.0001, ΔS = −2.22), ubiquitin (*p* < 0.0001, ΔS = −2.56), and PKA (*p* = 0.0005, ΔS = −11.48). The findings indicate that the tested compounds preferentially bind to OSM, suggesting that their docking scores are not due to non-specific interactions. The significant statistical difference between OSM and the control proteins strongly supports the validity of these binding interactions and helps eliminate the possibility of false-positive results.

### 2.5. Molecular Dynamics (MD) Simulations for Human Oncostatin M Using MOE

Root Mean Square Deviation (RMSD) is a widely used parameter in molecular dynamics simulations that describes the average displacement of atomic positions over time relative to a reference structure. Lower RMSD values (<3 Å) often indicate stable protein–ligand interactions, while higher or fluctuating RMSD values suggest conformational changes or instability [33].

**Figure 13:**
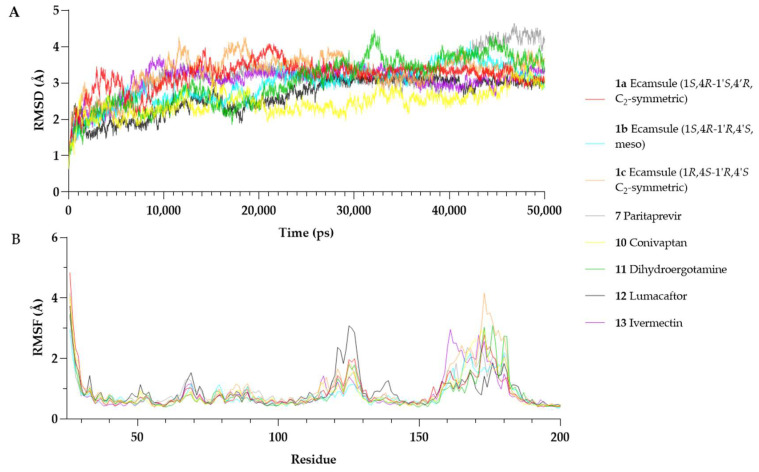
Molecular dynamics simulations over 50 ns. (**A**) Root Mean Square Deviation (RMSD) plots of oncostatin M in complex with different compounds. (**B**) Root Mean Square Fluctuation (RMSF) plots of oncostatin M residues over time.

The RMSD analysis showed that all OSM–compound complexes reached equilibrium within ~10–20 ns, with RMSD values stabilizing between 2.5 Å and 3.5 Å (Figure 13A). Conivaptan (**10**) exhibited the lowest RMSD (2.46 ± 0.35 Å), indicating a highly stable complex, followed by lumacaftor (**12**), the ecamsule isomer **1a** (3.2 ± 0.41 Å), the ecamsule isomer **1a** (2.97 ± 0.52 Å), and ivermectin (**13**) (3.06 ± 0.40 Å), which showed moderate stability. The RMSD of **1a** plateaued early (~10 ns) and remained stable with minor fluctuations (~0.3 Å), suggesting a well-fitted binding mode.

In contrast, some compounds showed more significant deviations. Dihydroergotamine (**11**) (3.03 ± 0.68 Å) fluctuated more than the others, likely due to conformational shifts. The ecamsule isomer (**1c**) exhibited a higher RMSD (3.29 ± 0.47 Å) and greater fluctuations, suggesting a suboptimal fit. Paritaprevir (**7**) (3.17 ± 0.61 Å) showed an increasing RMSD after ~30 ns, reaching ~4.0–4.2 Å by the end of the simulation, indicating possible destabilization.

Overall, conivaptan (**10**), lumacaftor (**12**), and the ecamsule isomer **1a** were the most stable binders, maintaining relatively low RMSD values. The ecamsule isomer **1c**, dihydroergotamine (**11**), and paritaprevir (**7**) exhibited greater fluctuations and reduced stability. The 50 ns simulation was sufficient for assessing binding stability in all cases except for paritaprevir (**7**), which showed a drift in the later stages.

Root Mean Square Fluctuation (RMSF) describes the positional variability in individual residues over time in a molecular dynamics simulation. Higher RMSF values indicate increased flexibility, while lower values suggest greater structural rigidity [34].

Figure 13 presents the per-residue RMSF profiles for each OSM–compound complex. Most structured regions of OSM exhibited low fluctuations (RMSF < 2 Å), confirming overall stability. However, residues Ser160–Pro180, which belong to the CD-loop region, showed increased flexibility across all simulations. This region is known for its structural mobility and has been reported to be involved in receptor interactions [27].

Among the tested compounds, the ecamsule isomer **1a** and conivaptan (**10**) appeared to stabilize the Ser160–Pro180 region, as their RMSF values remained within a range of 2.0–2.8 Å. Conversely, the ecamsule isomer (**1c**) and dihydroergotamine (**11**) exhibited greater flexibility (~3.5–4.2 Å), indicating weaker stabilization. Additionally, lumacaftor (**12**) increased fluctuations in Arg125 (~3.1 Å). This residue is positioned in helix C near the receptor-binding interface [27], potentially influencing OSM’s interaction with its receptor.

The MD simulations show that the ecamsule isomer **1a**, conivaptan (**10**), and lumacaftor (**12**) form the most stable interactions with OSM, as indicated by their relatively low RMSD values and reduced flexibility in functional regions. By contrast, the ecamsule isomer **1c**, paritaprevir (**7**), and dihydroergotamine (**11**) demonstrated increased structural fluctuations, which could influence their binding stability. However, as molecular dynamics simulations provide only a computational model of binding behavior, further in vitro studies are essential to validate these findings and assess their biological relevance.

### 2.6. Identification of Investigated Stereoisomer of Ecamsule as Structure **1a** by Nuclear Magnetic Resonance and Polarimetry

Virtual drug screening, molecular docking, and molecular dynamics simulation provided valuable insights into potential drug binding abilities, but the results obtained through in silico methods have to be confirmed by experimental validation. Given that ecamsule can occur in three stereoisomeric forms, the question arises as to which of the three possible stereoisomers was present in the commercially purchased ecamsule sample we used for further experiments.

Since no direct information was available from the provider regarding the stereochemical purity of the delivered ecamsule (**1**), specifically whether the sample was stereochemically homogeneous and, if so, which of the three possible stereoisomers it contained, a ^1^H NMR spectrum was measured in methanol-*d*_4_. These results showed a single set of signals (or, more precisely, half a set of signals, due to the symmetry of the structure) (Supplementary Figure S1). This clearly indicated that the sample was not a mixture of diastereomers but was either only the *meso* compound **1b** or only one of the two C_2_-symmetric enantiomers, **1a** or **1c**—or a racemic mixture of the two enantiomers, **1a** and **1c**.

The optical activity of the sample measured in methanol was +4.1°, indicating the absence of the optically inactive *meso* isomer, **1b**, or the racemic mixture of **1a** and **1c**. This positive value was in agreement with the likewise positive optical rotation of the synthetic starting material, 1*S*-(+) camphor sulfonic acid (+19.9°), which is also the commercially easily more available enantiomer (as the precursor for synthesis).

### 2.7. Microscale Thermophoresis of Oncostatin M Inhibitors

**Figure 14:**
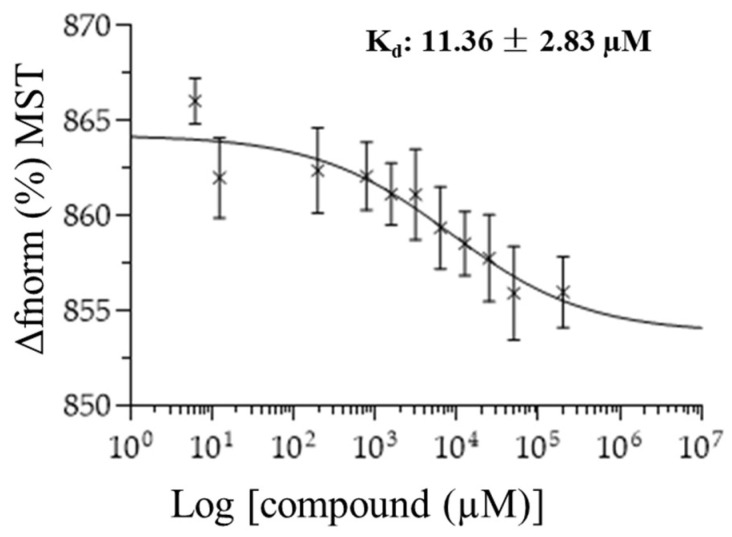
Concentration kinetics and dissociation constant (K_d_ value) of the ecamsule isomer 1a binding to oncostatin M as measured by microscale thermophoresis (n = 3).

We exemplarily investigated the binding of six selected compounds to oncostatin M using microscale thermophoresis as a sensitive assay. Recombinant human oncostatin M was utilized, and it was titrated against varying concentrations of the selected candidate compounds (Figure 14). The concentration kinetic curves of three independent experiments were used to calculate the dissociation constants (K_d_), which showed interactions between human oncostatin M and ecamsule (**1a**) (K_d_: 11.36 ± 2.83 µM). The raw data of MST curves are also available in Supplementary Figure S2 in three repetitions.

### 2.8. Quantitative Real-Time Reverse Transcription PCR of Selected Genes

**Figure 15:**
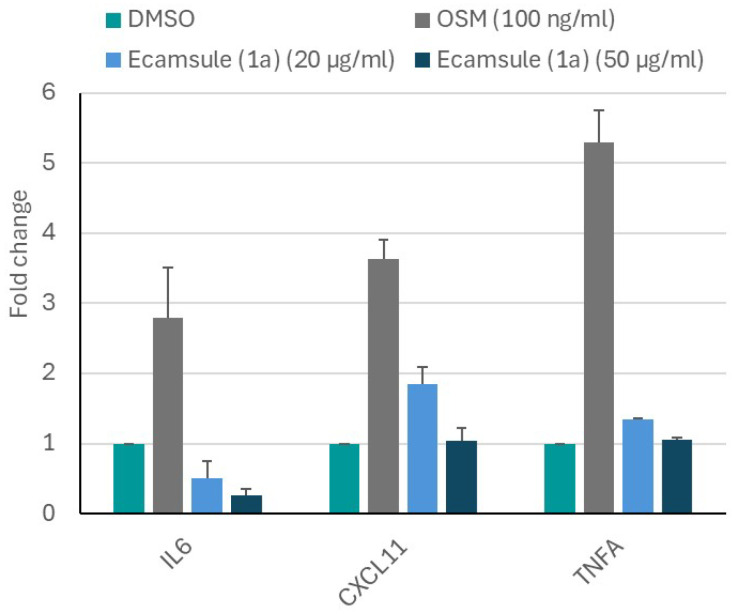
Inhibition of oncostatin M-stimulated mRNA expression of inflammatory cytokines and chemokines (*IL6*, *TNFA*, and *CXCL11*) by ecamsule (**1a**) as shown by qRT-PCR. Treatment of CaCo-2 cells with 100 ng/mL oncostatin M upregulated the expression of all three genes compared to the DMSO-treated control. However, pretreatment for 24 h with the ecamsule isomer 1a at concentrations of 20 and 50 µM reduced the activation of oncostatin M, resulting in the reduced expression of *IL6*, *TNFA*, and *CXCL11*.

While the microscale thermophoresis experiment clearly showed that ecamsule (**1a**) bound to oncostatin M, the question remains as to whether this binding exerts any functional effects. Therefore, we performed an in vitro experiment with CaCo-2 cells. We applied oncostatin M to Caco-2 cells and treated the cells with or without ecamsule (**1a**). We hypothesized that oncostatin M treatment may induce an inflammatory response, while ecamsule (**1a**) as a putative inhibitor would reverse the effect of oncostatin M. The results are shown in Figure 15. Oncostatin M treatment resulted in the upregulated mRNA expression of the inflammatory genes *IL6*, *TNFA*, and *CXCL1* compared to untreated control cells. 

However, pretreatment with ecamsule (**1a**) at concentrations of 20 and 50 µM notably reduced the oncostatin M-stimulated upregulation of these inflammatory biomarkers. This experiment indicates that ecamsule (**1a**) indeed has the capacity to reduce inflammatory responses induced by oncostatin M.

## 3. Discussion

Despite significant efforts to treat and cure Crohn’s disease, satisfactory pharmacological interventions are still not available. Therefore, the aim of the present investigation was to identify better drug targets and potential drug candidates.

The identification of drug targets has become increasingly important in the era of personalized and precision medicine. Numerous studies have focused on target identification and target-based drug discovery. Some notable strategies for discovering new targets that could be beneficial for drug development include the screening of large cell line panels with defined gene knockout using CRISPR/CAS9 technology [35,36], affinity-based pull-down methods [37,38], and the label-free targeting of molecules to specific targets [39,40], as well as “-omics” technologies combined with signaling pathway and network pharmacology analyses [41,42]. In recent years, computational methods utilizing virtual drug screening approaches have gained increasing attention [43,44].

In the current study, we employed a dual approach that combined transcriptomic mRNA sequencing with supercomputer-based virtual drug screening. These methods have previously been utilized by our team for different diseases [45,46]. To expedite the drug discovery process, we integrated both strategies in the current project.

Using intestinal biopsies from patients suffering from Crohn’s disease, we identified differentially expressed genes compared to colon biopsies from healthy individuals. Our analysis using Venn diagrams revealed six genes that were overexpressed in both ileum and colon samples from patients with Crohn’s disease compared to healthy samples. Genes that are commonly expressed in both the ileum and colon may serve as reliable markers for disease and could be targeted for treatment more effectively than those expressed in only one intestinal segment. These six genes may therefore encode possible targets to treat this disease, and they will be discussed in more detail.

*IL1B* encodes interleukin-1β, a classical pro-inflammatory cytokine, along with *IL6* (interleukin-6) and *TNFA* (tumor necrosis factor α), which are produced by monocytes and macrophages. Anti-inflammatory counterparts include IL1RA (interleukin-1 receptor antagonist), IL4 (interleukin-4), and IL10 (interleukin-10). An imbalance of pro-inflammatory cytokines can lead to acute intestinal inflammation progressing to chronic manifestation. Symptomatic (but not causative) treatments attempt to shift this disbalance toward anti-inflammatory regulation by cytokines to treat Crohn’s disease [47].

*TCL1A* encodes the T-cell leukemia/lymphoma 1A oncoprotein, which contributes to the development and progression of chronic lymphoblastic leukemia and lymphomas [48]. In cancer, this oncoprotein interacts with DNA repair deficiency, cell cycle arrest, apoptosis resistance, and overall genome instability. Its role in Crohn’s disease is not as well understood. The mechanisms relevant to cancer may also play a role in inflammatory diseases like Crohn’s disease by the modulation of immune responses and inflammatory processes.

The *HCAR3* gene codes for hydroxycarboxylic acid receptor 3, which acts as a membrane-bound G-protein-coupled receptor for lipid mediators that modulate the immune response [49]. It can influence the release of pro-inflammatory cytokines [50].

S100A8 activates neutrophil chemotaxis and contributes to an enhanced immune response and tissue damage in Crohn’s disease [51].

The *IGHG1* gene encodes the immunoglobulin heavy constant γ1 protein, which plays a role in humoral immune responses [52,53]. Its role in Crohn’s disease is not yet understood. IgG antibodies attack the intestinal tissue, and the consequently increased IGHG1 serum levels could contribute to inflammation [54].

In our analysis, *OSM* was found to be another gene that is overexpressed in the ileum and colon of patients with Crohn’s disease. *OSM* encodes oncostatin M, a member of the leukemia inhibitory factor/oncostatin M (LIF/OSM) family, which is a cytokine and growth regulator that controls the production of many other cytokines. This protein is known to be important in the pathogenesis of Crohn’s disease [54,55]. Furthermore, high levels of oncostatin M have been correlated with resistance to therapeutic monoclonal antibodies [56,57]. Therefore, we believe that oncostatin M is a suitable target for identifying drugs against Crohn’s disease that target this protein. Our perspective is supported by the fact that oncostatin M has been identified as a predictive biomarker for the outcome of Crohn’s disease [57,58]. Based on our results from transcriptomic analyses and evidence from the literature, we have chosen oncostatin M as our target of choice.

Furthermore, we did not only find that the *OSM* gene was overexpressed in the transcriptome analysis, but also confirmed through immunohistochemistry that oncostatin M protein was expressed in the Crohn’s disease biopsies. This is significant because the signaling to inflammatory reactions relies on the protein rather than the mRNA expression. Interestingly, oncostatin M was expressed not only in human biopsies but also in the colon of murine wild-type and knockout mice. Recently, kindlin-1 and kindlin-2 knockout mice have been used as experimental models to replicate the situation in human inflammatory bowel disease [23,24]. Therefore, these knockout mouse models may serve as suitable models to test the candidate drugs identified by us against Crohn’s disease.

We hypothesized that the pharmacological inhibition of oncostatin M and thus its downstream signaling route may represent a promising treatment strategy. To test this hypothesis, we have chosen a drug-repurposing approach. This concept refers to the idea that some drugs may be effective against not only the diseases they were originally approved for, but also other diseases, by targeting different pathways. There is a wealth of research on the off-label use and off-target effects of approved drugs. These effects can be beneficial if systematically investigated to find approved drugs for new treatment indications [59,60]. The drug-repurposing concept is considered promising because it offers several advantages over traditional drug discovery approaches. The safety and efficacy of approved drugs have already been established in previous studies. Therefore, the time and costs required to develop such a drug for a new disease will be significantly reduced. Furthermore, the side effects and toxicity profiles are known from previous studies, which accelerates the entire developmental process. The main actions of drugs against the diseases they were approved for may now be considered side effects if used for new indications. Nonetheless, drug repurpose is an attractive strategy to identify new treatment options for diseases that have been difficult to treat. We have successfully applied drug-repurposing strategies in the past to identify novel cancer treatments [61,62].

In the current project, we discovered several compounds with high binding affinities to oncostatin M. From these, we selected six drugs from our virtual screening approach that appeared most suitable for further verification. An exclusion criterion was the presence of fluorescing properties in some of the compounds. Fluorescing compounds can interfere with and falsify the results of test systems that rely on detecting fluorescent colors. This issue is known in the literature as the PAINS (pan-assay interference) phenomenon, which refers to pan-assay interference substances that often produce non-specific, false-positive results [63,64]. Because the experimental protocol for microscale thermophoresis involved the fluorescent labeling of recombinant target proteins, the fluorescing characteristics of drug molecules could impact the test outcomes. Therefore, sulfasalazine, commonly used to treat inflammatory bowel disease (IBD), was deemed unsuitable as a positive control. Adapalene, which exhibits blue fluorescence, was also excluded from the microscale thermophoresis experiments. We selected six drugs to confirm whether they bind to oncostatin M and found that all of them did, albeit with varying dissociation constants.

Ecamsule showed the best binding energy (LBE, pK_i_) in silico and the best dissociation constant (K_d_) in vitro. This firm binding arises from its very particular structure, unique among the 13 compounds investigated in this study: it is the only tested compound that has two highly acidic sulfonic acid elements. Acidic elements as such are not rare among the investigated structures, such as carboxylic acid residues in compounds **3**, **4** (here, even two such groups!), **5**, **6**, and **12** and also the less active, but still N,H-acidic, carbamide or lactam and sulfonamide functions in no less than seven of the compounds (**2**, **5**, **7**–**11**), all potential H-bridge donors—but none of them reach the high acidity of the two sulfonate groups in **1**. Another characteristic feature of **1** is its high symmetry (otherwise only present in compound **4**), the bulky bicyclic outer ring systems, with the center being flat and quite slim, and the large lipophilicity of the molecule, which contrasts with the polar sulfonate groups.

The toxicity profile of ecamsule shows that it is not linked with severe side effects (Table 4). However, the profile of side effects for ecamsule as a topical sun protectant might differ from that of oral use, which is the most probable application route if this compound is to be further developed for the treatment of Crohn’s disease. It is also of significance whether the drug is absorbed or—most likely—remains in the intestinal lumen to exert its anti-inflammatory efficacy. Thus, the toxicity profile for the oral application route is still elusive.

Interestingly, ecamsule can occur in three different stereochemical forms—two C_2_-symmetric enantiomers and a *meso* form. The substantial relevance of these stereochemical issues was evidenced already in the docking experiments, where the three stereoisomers showed slight but significant differences in the lowest binding energies, as determined by AutoDock (see Table 1). Accordingly, it seems that the 1*S*,4*R* half is more tightly bound than the 1*R*,4*S* portion. This makes understandable the finding that **1a**, which has two such 1*S*,4*R* moieties (1*S*,4*R*-1′*S*,4′*R*), binds more strongly (by 0.11 kcal/mol) to oncostatin M than **1b**, which has only one such better-fitting 1*S*,4*R* part (1*S*,4*R*-1′*R*,4′*S*). And **1c**, in turn, which has no such favorable 1*R*,4*S* entity (1*S*,4*R*-1′*S*,4′*R*), binds even less strongly than **1b**, here even by another 0.24 kcal/mol, showing the significant influence of the smallest stereochemical details on docking behavior.

The stereoisomer-specific docking energies can be convincingly substantiated by looking at the respective amino acids found to bind to the individual stereoisomers of ecamsule (compounds **1a**, **1b**, and **1c**). In compound **1a**, which consists of two identical chiral building blocks, both 1*S*,4*R*, one half is bound to five amino acids, ARG52, ALA55, PHE56, ARG84, and PHE169, while the other is bound to only three such amino acids, SER157, ALA158, and ARG162. Like the symmetric **1a**, the *meso* compound **1b** is also bound to exactly those same five amino acids, ARG52, ALA55, PHE56, ARG84, and PHE169, but only in one moiety, indicating that this must be the 1*S*,4*R* portion of **1b**; this shows that this is a strong interaction, fully conserved in the transition from **1a** to **1b**, while the other part of **1b**, the 1′*R*,4′*S* entity, has to find amino acids fitting with its different configuration, namely, ALA156, SER157, and ALA162. This means a small change of just one amino acid (out of a total of eight) in the transition from **1a** to **1b**. Much more dramatic, accompanied by a change of three interacting amino acids in total, is the transition from **1b** to **1c**. In **1c**, the 1*R*,4*S* moiety (instead of 1*S*,4*R* in **1b**) has only four remaining amino acids, ARG52, ALA55, ARG168, and PHE169; one is changed, and one is lost. Even the less drastic structural change from the 1*R*,4*S* entity in **1b** to the formally identical 1*R*,4*S* part in **1c** is accompanied by a change of one amino acid, ARG91. This clarifies that the binding properties of **1a** and **1b** are more similar to each other than those of **1b** to **1c**, as reflected by the energy difference of 0.11 kcal/mol versus 0.24 kcal/mol.

In view of the strongest-binding property of **1a** among the ecamsule isomers, it was thus a fortunate circumstance that we found out that the commercially available, moreover, chemically pure (98%), ecamsule material was the C_2_-symmetric isomer **1a**—and that it was stereochemically homogeneous.

Conivaptan (**10**) seems to be another considerable candidate for the treatment of Crohn’s disease since the side effects of this drug are mild and manageable (Table 4).

Ivermectin (**13**) demonstrates anti-inflammatory features by inhibiting the production of pro-inflammatory cytokines [65]. Furthermore, ivermectin acts in an anti-inflammatory manner in experimentally induced colitis in rats [66]. Therefore, we added ivermectin to the list of 13 compounds that were tested in silico and in vitro, although it did not appear among the top 10 compounds identified by molecular docking. We found that ivermectin (**13**) indeed binds to oncostatin M, albeit only with a mediocre affinity, in microscale thermophoresis.

Simeprevir (**2**) and paritaprevir (**7**) are probably less suited for the treatment of Crohn’s disease since they are too expensive for routine use and can exert hepatotoxicity in some cases. Venetoclax (**8**), indacaterol (**9**), dihydroergotamine (**11**), and lumacaftor (**12**) also exert considerable adverse effects. Telmisartan (**3**) is a standard drug against hypertension. However, it has been recently called into question whether or not it might increase carcinogenic risk [67]. Therefore, we did not conduct further follow-up studies on this compound. Having performed a bioinformatically based screening, we identified candidates out of a chemical library of 1577 FDA-approved drugs that might be useful to treat Crohn’s disease. Among the long list of compounds, we favor ecamsule (**1a**) and conivaptan (**10**) as interesting candidates that are worth exploring in terms of their utility in clinical trials.

The question arises as to whether other authors have also applied drug-repurposing approaches to identify FDA-approved drugs as novel candidates for the treatment of Crohn’s disease. Kwak and colleagues [68] performed single-cell network-based drug repositioning and identified vorinostat as a potential drug candidate for patients with anti-TNF-resistant Crohn’s disease. Vorinostat is a drug used to treat T-cell lymphoma, and it exerts many of the severe toxicities known to be exerted by many anticancer drugs. A Danish study using real-world data took advantage of health files to correlate drug usage with an increased or reduced risk of fibrosis in patients with Crohn’s disease [69].

As there is no direct functional assay available for oncostatin M, we decided to monitor inflammatory downstream genes that are activated by oncostatin M, i.e., *IL6*, *TNFA*, and *CXCL1*. The OSMβ receptor and its co-receptor glycoprotein 130 (gp130) activate several signal transduction pathways upon the binding of oncostatin M, including JAK/STAT, MAPK/ERK, and PI3K/AKT/mTOR [70,71]. The activation of these pathways ultimately leads to the expression of numerous cytokines and chemokines [70]. Our qRT-PCR results demonstrated that preincubation with ecamsule (**1a**) apparently inhibited binding to its receptors and thereby suppressed the expression of *IL6*, *TNFA*, and *CXCL1*. We conclude that ecamsule (**1a**) inhibits inflammation by inhibiting oncostatin M signaling. Therefore, ecamsule (**1a**) may be a valuable candidate drug for the treatment of Crohn’s disease.

Independent of drug-repurposing approaches, other small molecules and therapeutic monoclonal antibodies targeting oncostatin M have been recently described [72,73]. Whether they are more useful for the treatment of cancer or whether they may also be applicable to the treatment of Crohn’s disease and how they compare with ecamsule (**1a**), as suggested by us in the present investigation, remain to be clinically studied in the future.

## 4. Material and Methods

### 4.1. mRNA Isolation from Human Intestinal Biopsies

mRNA isolation from the intestinal tissue biopsies was carried out under sterile conditions using nuclease-free pipette tips and the RNeasy mini kit (Qiagen, Hilden, Germany) according to the manufacturer’s instructions. In brief, 20 to 30 mg of tissue was first homogenized in 350 µL RLT lysis buffer in the TissueLyser (Qiagen, Hilden, Germany) at 2000 rpm for 20 s. The lysate was then transferred to a 1.5 mL Eppendorf tube and centrifuged at maximum speed for 4 min. The supernatant was carefully decanted into a new Eppendorf tube, and the pellet was discarded. As a next step, 1× volume (corresponding to 350 µL) of 70% ethanol was added to the lysate. The total volume was quickly mixed with a pipette, applied to an RNeasy spin column, and centrifuged at 8000× *g*. The RNA bound to the column membrane and the flow was discarded. Then, three washing steps followed: (1) with 700 µL RW1 buffer at 8000× *g* for 15 s, (2) with 500 µL RPE buffer for 8000× *g* for 15 s, and (3) with 500 µL RPE buffer 8000× *g* for 2 min.

In order to completely remove buffer residues, the column was placed in a new collection tube and centrifuged dry at maximum speed for 1 min. The spin column was then placed in a 1.5 mL Eppendorf tube, and the RNA was eluted with 50 µL of RNase-free water by centrifugation at 8000× *g* for 1 min. To increase yield, the water was allowed to incubate on the membrane for 1 min prior to elution.

The concentration was determined using the NanoDrop ND 1000 UV-Vis spectrophotometer (Thermo Fisher Scientific, Dreieich, Germany). Sample aliquots of 1.5 µL were applied to the measurement field without any air bubbles. Samples were washed with deionized water before and after measurement. The zero adjustment, blank, was carried out with the elution solution of the sample. The samples were immediately placed on dry ice and stored at −80 °C.

### 4.2. Determination of RNA Integrity

The verification of RNA integrity was carried out using an Agilent 2100 Bioanalyzer (Agilent Technologies, Santa Clara, CA, USA) using commercial kits: the Agilent 2100 Nano Kit for RNA concentrations ranging from 25 to 500 ng/µL and the Agilent 2100 Pico Kit (Agilent Technologies, Santa Clara, CA, USA) for concentrations from 50 to 5000 pg/µL. For all other concentrations, the RNA was appropriately diluted with deionized water. The principle of the analysis is based on RNA. The chip format of the assay is based on agarose gel electrophoresis with a significantly reduced sample volume and separation time. The procedure was carried out according to the instructions of the manufacturer. Briefly, 550 µL of the gel matrix was pipetted onto a spin filter and centrifuged for 10 min at 1500× *g* at room temperature. For each 65 µL aliquot, 1 µL of the RNA 6000 Nano dye was added, mixed thoroughly, and centrifuged for 10 min at 13,000× *g* at room temperature. In order to load the chip with the gel–dye mix, it was placed in the corresponding priming station. An aliquot of 9 µL of the prepared gel was then pipetted into a marked well. The capillaries in the chip were filled with the gel–dye mix under pressure. Two additional wells were also loaded with 9 µL gel. The marker (5 µL) was pipetted into all sample wells, including the well with the laddered size marker. The marker was used to align the ladder data with the sample data and to compensate for any drift effects that may have arisen during the analysis. Subsequently, 1 µL of the size marker and 1 µL of the RNA samples were pipetted into the corresponding wells.

Finally, the chip was vortexed at 2400 rpm and immediately measured in the Agilent 2100 Bioanalyzer. In addition to the RNA integrity, concentration was also determined at the same time. During analysis, the dye molecules intercalated into the RNA strands. These complexes were separated electrophoretically according to their size and detected by laser-induced fluorescence. The result was displayed as an electropherogram and as a virtual gel image. Using the ratio of the 285 rRNA and the 185 rRNA, the software determined an RNA integrity number (RIN) from 1 to 10. A value of 10 represented optimal RNA integrity without any degradation, whereas a value of 1 indicated a completely degraded RNA sample. The ideal ratio was 2.0. In addition, other signals, such as intermediate peaks, also played a role in determining the RIN.

### 4.3. cDNA Synthesis by Reverse Transcription

RNA was transcribed into cDNA by reverse transcription. The cDNA synthesis was performed on ice using the RevertAid First Strand cDNA Synthesis Kit (Thermo Fisher Scientific, Dreieich, Germany). The RNA-dependent DNA polymerase first transcribed the RNA into a single-stranded cDNA. A random hexamer primer hybridized to the mRNA, which was then extended by reverse transcriptase. The cDNA first strand synthesized in this way served as a template to be amplified by the subsequent PCR. Aliquots of 1.5 µg RNA were used per sample. After 1 µL of primer was added, the final volume was filled up to 12 µL with RNase-free water. The samples were then denatured at 65 °C for 5 min. To prevent the regression of secondary structures, the sample tubes were placed directly back on ice. The further reaction components were then added in the following order: 1.4 µL 5× reaction buffer, 1 µL RiboLock™ RNase Inhibitor (20 U/μL), 2 µL 10 mM dNTP Mix, and 1 µL RevertAid H Minus M-MuLV Reverse Transcriptase (200 U/µL).

The mixtures were carefully mixed and centrifuged. After incubation at 25 °C for 5 min, synthesis was carried out at 42 °C for 1 h in a thermomixer. To inactivate the reverse transcriptase, the mixtures were heated at 70 °C for 5 min. A reverse transcriptase negative control (RT–) was established for each RNA sample, where water was added instead of reverse transcriptase. This control served to exclude genomic contamination. In addition, a no-template negative control (NTC control) was included, where the RNA was replaced with water to detect the possible contamination of reagents.

### 4.4. One-Color Microarray Gene Expression Analysis

RNA from Crohn’s disease biopsies and healthy colon and ileum samples (n = each) was converted to cDNA and labeled by adding fluorescently labeled nucleotides in reverse transcription. Single-stranded oligonucleotide sequences with a length of 60 nucleotides, anchored on a solid support, were hybridized with our cDNA samples at 65 °C for 17 h. We used the Agilent Oligo Microarray Kit (Agilent Technologies, Santa Clara, CA, USA) for this process, where the binding occurred specifically via hydrogen bonds. Non-specifically bound nucleotides were removed from the oligonucleotide microarray through several washing steps. The subsequent scanning process compared the transcription levels of healthy and diseased tissues, with the evaluation initially carried out using bioinformatic methods at the Institute of Molecular Biology (Mainz, Germany).

The first step involved preparing the one-color spike mix. For this purpose, the stock solution was vortexed and heated at 37 °C for 5 min. The solution was then serially diluted (1:20, 1:500, 1:10,000) with dilution buffer. The first dilution (1:20) was stored at −80 °C for two months and could be frozen and thawed up to eight times. Next, 100 to 200 ng of total RNA, brought to a volume of 1.5 μL and 2 μL of the 1:10,000 dilution, was added. After adding 1.8 µL of the 17 Promoter Primer Mix, the mixtures were denatured at 65 °C for 10 min and then immediately placed on ice. For cDNA synthesis, 2 µL of 5× First Strand Buffer (preheated), 1 µL 0.1 M dithiothreitol (DTT), 0.5 µL of 10 mM dNTP Mix, and 1.2 µL of AffinityScript RNase Block Mix were added to each batch and mixed by pipetting up and down.

The mixtures were incubated at 40 °C for 2 h. The reverse transcriptase was then inactivated by heating at 70 °C for 15 min. For fluorescence cRNA synthesis, 0.75 µL of water (nuclease-free), 3.2 µL of 5× transcription buffer, 0.6 µL of 0,1 M DTT, 1 µL of dNTP Mix, 0.21 µL of T7 RNA Polymerase, and 0.24 µL of cyanine 3-CTP reagents were pipetted into each batch.

The synthesis was carried out protected from light at 40 °C for 2 h. Subsequently, the cRNA was purified using the Qiagen RNeasy Kit (Qiagen, Hilden, Germany). The cRNA was brought to a volume of 100 µL with nuclease-free water, and then 350 µL of RLT buffer and 250 µL of 100% ethanol were added. The mentioned reagents were filled in an RNeasy mini column and centrifuged at 13,000 rpm for 30 s. The supernatant was discarded, and 500 µL RPE buffer was added. The column was centrifuged at 13,000 rpm for 30 s, and the flowthrough was discarded. Afterwards, we washed again with 500 µL RPE buffer and eluted the cRNA with 30 µL nuclease-free water. The quantification of the cRNA was performed using NanoDrop. The calculated yields were greater than 825 ng. In addition, 5 µL 10× blocking reagent in a volume of 24 µL of nuclease-free water and 1 µL of 25× fragmentation buffer was added to 825 ng of cyanine-3-labeled, linearly amplified RNA.

The mixture was incubated at 60 °C for exactly 30 min and then immediately placed on ice. To stop the reaction, 25 μL of 2× HiRPM hybridization buffer was pipetted in and mixed carefully. The gasket slides were immediately loaded and hybridized in the hybridization chamber in the oven at 65 °C and 10 rpm for 17 h. The slides were washed the following day, with one chamber filled with Gene Expression Wash Buffer 1 and another chamber with 37 °C Gene Expression Wash Buffer 2. The temperature was kept constant using a heating stirrer. The slides were removed from the hybridization chamber and washed for 1 min each. Finally, the slides were placed in a holder and scanned immediately. The detection of the fluorescence signals was based on the principle of confocal laser scanning microscopy, where two lasers with corresponding wavelengths were used to scan the arrays with photomultipliers serving as detectors.

For evaluation, the data were visualized and sorted using Chipster software (http://chipster.csc.fi/) (version3.16.3) (accessed on 21 June 2020) to sort out the affected genes according to their variable for expression and significance based on the empirical Bayes *t*-test (*p* < 0.05). The genes were further analyzed using Ingenuity Pathway Analysis software (content version: 51963813; release date: 11 March 2020) (IPA; Ingenuity Systems, Redwood City, CA, USA).

### 4.5. Immunohistochemistry

Colon and ileum biopsies from patients with Crohn’s disease and healthy control tissues from the colon and ileum were collected between 2001 and 2008. The samples were obtained by two authors: W.S. (from the Department of Gastroenterology and Hepatology, University Hospital of Heidelberg, Heidelberg, Germany) and W.R. (from the Institute of Pathology, University Medical Center, Johannes Gutenberg University, Mainz, Germany). The Ethical Committees of the collecting centers approved the protocol, and written informed consent was obtained from all patients. Ethical approvals were granted by the Ethics Committee of the University of Heidelberg to W.S. (EC-L69/2003 and EC-L71/2003) and by the Ethics Committee of the State Authorization Association for Medical Issues (Landesärztekammer Rheinland-Pfalz) to W.R. (2 October 2015; Ref. 837.031 9799) and to T.E. (22 March 2018; Ref. 2018-13179). Participants provided their approval for the evaluation of tissues and the publication of data generated from these investigations prior to participation.

Furthermore, colon and ileum biopsies of kindlin-1 and -2 intestine-specific knockout mice were collected. Animal studies followed the “ARRIVE” guidelines and were approved by the Heidelberg Ethics Committee [Ref # 35–9185.81/6123/10 and 6284/11] as well as the acquisition of human biopsy samples [Ref # S-211/2010]. Male C57BL/6 wild-type mice were obtained from Charles River Laboratories (Göttingen, Germany). The corresponding tamoxifen-inducible, villin-Cre-dependent, kindlin-1 and -2 intestine-specific knockout mice were gifts from Reinhard Fässler (Department of Molecular Medicine, Max Planck Institute of Biochemistry, Martinsried, Germany) [23], with samples propagated after embryonic transfer in the animal facilities of the University of Heidelberg. Wild-type and mutant mice were co-housed in the sense that wild-type mice and kindlin-1^−/−^ and kindlin-2^−/−^ mice were kept under identical breeding conditions in the same environment as previously described [24].

The tissues were fixed in paraffin and stored at room temperature. Paraffin-embedded tissues were cut into 5 µm thick sections using a microtome in the Department of Pharmaceutical Biology, JGU, Mainz, Germany. A detailed step-by-step protocol of the immunohistochemical procedure has been recently published by our group [74]. In brief, a micro polymer labeling technique was applied (mouse- and rabbit-specific horseradish peroxidase (HRP)/3,3’-diaminobenzidine (DAB) IHC Detection Kit, ab236466; Abcam, Munich, Germany), including hydrogen peroxide block, protein block, mouse-specifying reagent (complement), goat-anti-rabbit HRP-conjugate, DAB chromogen, and DAB substrate. The primary antibody used was oncostatin M (type II) polyclonal antibody (dilution 1:100; invitrogen, Europe, Darmstadt, Germany; Catalog #:PA5-76861) generated against recombinant human oncostatin M/OSM protein and verified by HPLC.

Chromogen detection was performed with DAB reagent, and slides were counterstained with hematoxylin. For the negative control, the primary antibody was omitted, and antibody diluent was used instead. For assessment, the immunostained slides were scanned by Panoramic Desk.

### 4.6. Virtual Screening and Molecular Docking

A total of 1577 FDA-approved compounds were downloaded from the ZINC database (https://zinc15.docking.org/, accessed on 28 February 2018). The 3D structures of our ligands were downloaded as standard data files (sdf). Subsequently, we utilized https://cactus.nci.nih.gov/translate/ (accessed on 28 February 2018) to convert the structure of our ligands from “sdf” to “pdb”. Next, we employed AutoDockTool 1.5.6 to convert our FDA-approved ligands from “pdb” to “pdbqt”. The crystal structure of oncostatin M was downloaded as a “pdb” file (PDB ID: 1EVS) from the Protein Data Bank (https://www.rcsb.org/structure/1EVS accessed on 28 February 2018). The protein crystal structure was processed by deleting water molecules and adding missing hydrogen atoms. The data structure was ultimately saved in “pdbqt” format. We subsequently performed blind docking using V0.8 virtual screening software (Version 0.8). Available online: https://pyrx.sourceforge.io/ (accessed on 31 March 2023)

Based on the lowest binding energies (kcal/mol), i.e., the highest binding affinities, we chose the top 50 compounds for molecular docking analyses with AutoDock 1.5.6. (https://autodock.scripps.edu/, La Jolla, CA, USA) using a defined docking approach. The grid box for defined docking was set on the whole A-chain of oncostatin M with the center of the grid box at the following coordinates: x = 14.559, y = 35.559, and z = 30.586, with the number of grid points (ntps) being 74 in x, 60 in y, and 106 in z with spacing of 0.375. The active site of oncostatin M chosen for grid box localization was taken from the Computed Atlas of Surface Topography of Proteins (CASTp) [75]. OSM-SMI8 has been described as a synthetic inhibitor of oncostatin M [76]. The IUPAC name is as follows: (2*Z*,4*E*)-2-[2-[4-[(1*Z*,3*E*)-1-carboxy-4-phenylbuta-1,3-dienyl]-1,3-thiazol-2-yl]-1,3-thiazol-4-yl]-5-phenylpenta-2,4-dienoic acid (CAS No.: 1689690-20-7). OSM-SMI8 served as a positive control drug. Then, the AutoDock-based calculation was performed using a Lamarckian algorithm run with 250 runs and 2,500,000 energy evaluations. The AutoDock RMSD cluster analysis generated the expected results as DLG files. After picking the grid box and preparing the docking parameter files, the docking procedure was carried out using the MOGON supercomputer and the advisory services provided by Johannes Gutenberg University Mainz (hpc.uni-mainz.de). Johannes Gutenberg University Mainz is a member of the AHRP (Alliance for High-Performance Computing in Rhineland-Palatinate) and the Gauss Alliance e.V.

The figures were prepared using Visual Molecular Dynamics (VMD). For the visualization of the molecular docking results, we used the BIOVIA Discovery Studio Visualizer (https://discover.3ds.com/ accessed on 28 February 2018) to determine the interactions of the candidate ligands with the corresponding amino acids of oncostatin M in 3D and 2D modes.

### 4.7. Molecular Docking with MOE

For molecular docking studies, the recently published crystal structure of human oncostatin M (PDB Code 8V29) was utilized instead of the previously available structure (PDB ID: 1EVS). The protein was prepared using the QuickPrep function in Molecular Operating Environment (MOE) software version 2022.02 from the Chemical Computing Group (https://www.chemcomp.com, accessed on 8 December 2024). This function includes energy minimization with the Amber10:EHT force field, the protonation of residues, and the repair of any missing residues and atoms. Additionally, a missing loop spanning from Ser160 to Pro178 was rebuilt using the De Novo Loop Modeler function in MOE. A total of 1000 loop conformations were generated, and the loop with total sequence identity and the best score values was chosen for further analysis.

The tested compounds were prepared using the wash-dominant mode at pH 7 in MOE to ensure correct protonation states. Before docking, energy minimization was performed using the Amber10:EHT force field.

Docking simulations were conducted using the General Dock module in MOE. The Triangle Matcher method was employed for initial placement, followed by Induced Fit refinement. The scoring function London dG [77] was applied during placement, while GBVI/WSA dG [77] was used to rescore refined poses. Each compound was docked in three independent docking runs, with 100 initial poses and 10 refined poses per run. The final docking scores (S) and RMSD refine values were averaged across replicates, and standard deviations were calculated.

To assess the specificity of compound binding, docking was also performed against three negative control proteins: keratin (PDB ID: 6EC0), ubiquitin (PDB ID: 8ST7), and protein kinase A (PKA, PDB ID: 1ATP). The same QuickPrep procedure was applied for protein preparation. For keratin and ubiquitin, the entire protein was chosen as the docking site, while the ATP-binding site was selected for PKA. Docking parameters remained consistent with those used for OSM to ensure comparability.

To statistically assess the specificity of compound binding, a one-way ANOVA with Welch’s correction was conducted using GraphPad Prism 8 (GraphPad, La Jolla, CA, USA). Docking scores from three independent docking runs were compared for oncostatin M against three negative control proteins. The Brown–Forsythe and Welch ANOVA tests were chosen to address unequal variances. Post hoc comparisons were performed using the Games–Howell multiple comparisons test, which does not presuppose equal standard deviations. The family-wise significance level was set at α = 0.05, and multiplicity-adjusted *p*-values were provided for each comparison.

### 4.8. Molecular Dynamics (MD) Simulations

Molecular dynamics (MD) simulations were conducted to investigate the stability and interaction dynamics of the protein–ligand complexes, which were identified through prior molecular docking studies using MOE 2022.02. Each compound was docked in three independent docking repetitions. The docking pose with the best docking score was selected for further MD simulations.

Energy minimization and protonation were performed via the QuickPrep function in MOE, using the Amber 10:EHT force field. The prepared protein–ligand complexes were then solvated and further processed for MD simulations using NAMD 2.14 MacOSX-x86_64 [75]. After energy minimization, an equilibration was carried out in two phases. The system was initially heated to 300 K over 100 ps in the NVT ensemble (constant number of particles, volume, and temperature). This was followed by equilibration in the NPT ensemble (constant number of particles, pressure, and temperature) for 200 ps at 300 K and 1 atm. The production run was carried out for 50 ns under NPT conditions.

Trajectory analysis was performed using VMD version 1.9.4a53 [76]. Structural deviations and fluctuations were assessed by calculating Root Mean Square Deviation (RMSD) and Root Mean Square Fluctuation (RMSF) throughout the simulation. All simulations were conducted under periodic boundary conditions with explicit solvation to ensure a realistic dynamic environment for the studied complexes.

### 4.9. Nuclear Magnetic Resonance and Optical Activity of Ecamsule

The nuclear magnetic resonance (NMR) spectrum of commercially acquired ecamsule (**1a**) (Sigma-Aldrich, Steinheim, Germany; purity: ≥98%) was measured on a Bruker Avance III HD 600 (600 MHz) instrument (Bruker, Karlsruhe, Germany), using methanol-*d*_4_ (*δ*_H_ = 3.31 ppm) as the solvent. The spectrum was acquired and processed using an ACD/NMR processor (version 12.01) and Topspin 3.2 software (Bruker Daltonics, Billerica, MA, USA). The optical rotation of **1a** was +4.1°, determined at a wavelength of 589 nm on an Anton Paar polarimeter (cell length 100 mm) in methanol as the solvent.

### 4.10. Microscale Thermophoresis

For microscale thermophoresis, we purchased ecamsule (**1a**) (Sigma-Aldrich, Steinheim, Germany, purity ≥98%). We determined the dissociation constants (K_d_) for the binding of ecamsule (**1a**) to recombinant human oncostatin M (Sino Biological, Eschborn, Germany Catalog # 10452-HNAH). The recombinant protein was labeled with Monolith Protein Labeling Kit RED—NHS 2nd Generation (MO-L011, Nano Temper Technologies, Munich, Germany) following the manufacturer’s instructions. The final protein concentration after labeling was 1000 nM in all repetitions. The titration was carried out using a wide concentration range of the compounds (dilution steps 1:1). The DMSO concentration in all the different ligand concentrations was stable (2.5%). The ligand and protein were incubated for 30 min at room temperature in assay buffer (50 mM Tris buffer (pH 7.4) containing 10 mM MgCl_2_, 150 mM NaCl, and 0.05% Tween-20). Measurements were carried out in Monolith NT.115 standard capillaries (MO-K022, Nano Temper Technologies, Munich, Germany). The signals were measured using a Monolith NT.115 instrument (Nano Temper Technologies, Munich, Germany) with 20% light-emitting diode (LED) power with 10 MST for ecamsule (**1a**). Fitting curves and K_d_ values were calculated using the MO 2022.02 AffinityAnalysis software (Nano Temper Technologies, Munich, Germany). The experiment was repeated three times.

### 4.11. Cell Culture

The human colon adenocarcinoma cell line Caco-2 was provided by the University Medical Center Mainz, Germany. The cells were routinely passaged and sustained in a humidified environment at 37 °C with 5% CO_2_. Caco-2 cells were consistently grown in MEM media enriched with 15% FBS and 1% penicillin–streptomycin (100 U/mL—100 μg/mL) (Invitrogen/Life Technologies, Darmstadt, Germany), 1% sodium pyruvate solution (100 mM in 100 mL) (Sigma-Aldrich, Taufkirchen, Germany), 1% non-essential amino acid solution 100× (Sigma-Aldrich, Taufkirchen, Germany), and Glutamax 100× (Thermo Fisher Scientific Gibco, Dreieich, Germany). Caco-2 cells cultured for 21 d develop monolayers of differentiated intestinal enterocytes [78]. Caco-2 cells were cultured in 6-well plates at an initial density of 25 × 10^4^ cells per well, with the culture media changed every other day. They were allowed to attain confluence and used for tests after 21 d to confirm complete differentiation. For all experiments, we utilized only cells in the exponential proliferation phase. The qPCR experiments were conducted on cells with passage numbers ranging from 20 to 50.

Differentiated Caco-2 cells were pretreated with 100 ng/mL recombinant oncostatin M (Sino Biological, Europe, Eschborn, Germany) for 24 h and then with or without 20 or 50 nM ecamsule (**1a**) (Sigma-Aldrich, Taufkirchen, Germany) for another 24 h. DMSO-treated cells served as a control. This experiment was repeated 3 different times.

### 4.12. Quantitative Real-Time Reverse Transcription PCR

The LunaScript^®^ RT SuperMix kit (New England Bio Laboratories, Frankfurt, Germany) was employed to extract cDNA from 1 μg of RNA. A NanoDrop 1000 spectrophotometer (PEQLAB, Erlangen, Germany) was used to measure the total RNA concentration post-extraction. Gene amplification was conducted using 5× Hot Start Taq EvaGreen qPCR Mix (Axon-Labortechnik, Kaiserslautern, Germany) according to the manufacturer’s guidelines. The primer pairs for the *IL6*, *TNFA*, and *CXCL11* genes were acquired from Eurofins Genomics (Ebersberg, Germany) via the NCBI/Primer-BLAST tool (https://www.ncbi.nlm.nih.gov/tools/primer-blast/) (accessed on 2 January 2025) after a comprehensive validation procedure. Primers have been identified for *IL6*, *GAPDH*, *CXCL11*, and *TNFA* (Table 5). The qRT-PCR reactions were conducted with a CFX384 instrument (Bio-Rad, Munich, Germany) with the following parameters: denaturation at 95 °C for 15 s, gradient annealing from 61.5 to 57 °C for 30 s, and elongation at 72 °C for 1 min. The Cq values were derived using Manager software (version 3.1) from Bio-CFX Rad. The changes in gene expression were assessed using the 2ΔΔCt methodology subsequent to adjusting gene expression to the expression of the control GAPDH in the relevant samples.

## Figures and Tables

**Table 1:** Virtual drug screening and molecular docking of 12 selected FDA-approved drugs binding to oncostatin M. OSM-SMI8 served as positive control drug. Virtual drug screening was performed using PyRx and molecular docking using AutoDock 1.5.6. Values for the lowest binding energies (LBEs) and predicted inhibition constants (pK_i_) are shown.

Drug	PyRx LBE (kcal/mol)	AutoDock LBE (kcal/mol)	AutoDock pK_i_ (nM)
**1a** Ecamsule (1*S*,4*R*-1′*S*,4′*R*, C_2_-symmetric)	−7.60	−10.46 ± 0.08	21.62 ± 2.97
**2** Simeprevir	−8.00	−10.39 ± 0.27	26.89 ± 13.33
**1b** Ecamsule (1*S*,4*R*-1′*R*,4′*S*, *meso*)	−7.50	−10.35 ± 0.39	31.15 ± 14.79
**1c** Ecamsule (1*R*,4*S*-1′*R*,4′*S*, C_2_-symmetric)	−7.60	−10.10 ± 0.09	40.18 ± 5.74
**3** Telmisartan	−8.00	−10.06 ± 0.03	42.27 ± 2.32
**4** OSM-SMI8 (control drug)	−7.30	−10.04 ± 0.12	44.82 ± 9.52
**5** Sulfasalazine	−8.00	−9.68 ± 0.01	80.30 ± 0.56
**6** Adapalene	−8.20	−9.56 ± 0.17	85.08 ± 7.41
**7** Paritaprevir	−8.50	−9.47 ± 0.07	114.57 ± 13.76
**8** Venetoclax	−7.90	−9.41 ± 0.17	131.18 ± 40.12
**9** Indacaterol	−8.00	−9.36 ± 0.14	142.25 ± 34.24
**10** Conivaptan	−8.80	−9.15 ± 0.04	196.83 ± 13.09
**11** Dihydroergotamine	−8.40	−9.05 ± 0.09	294.68 ± 64.70
**12** Lumacaftor	−8.94	−8.77 ± 0.06	373.78 ± 36.78
**13** Ivermectin	−8.80	−6.65 ± 0.30	15,740 ± 9274.24

**Table 2:** Molecular docking results for eight tested compounds with human oncostatin M (PDB ID: 8V29) using MOE 2022.02. The table shows the docking scores, S, with standard deviations, calculated from three independent docking runs, along with the RMSD refine values to further assess the quality of the docking poses. The amino acids interacting with the drugs were determined from the docking pose with the lowest docking score among the three runs, using a 4.5 Å cutoff.

Drug	Docking Score S	RMSD Refined	Amino Acids Interacting with the Drugs
**1a** Ecamsule (1*S*,4*R*-1′*S*,4′*R*, C_2_-symmetric)	−7.89 ± 0.02	1.65 ± 0.01	Arg77, Pro78, Gly79, Ala80, Pro82, Thr98, Ala101, Thr102, Cys105, Arg109, Lys167, Ala168, Arg170, Gly171, Glu190, Arg193, Phe194
**1b** Ecamsule (1*S*,4*R*-1′*R*,4′*S*, *meso*)	−7.95 ± 0.26	1.97 ± 0.20	Arg77, Pro78, Gly79, Ala80, Pro82, Gln97, Thr98, Ala101, Thr102, Cys105, Arg109, Lys167, Ala168, Arg170, Gly171, Ala172, Gln174
**1c** Ecamsule (1*R*,4*S*-1′*R*,4′*S*, C_2_-symmetric)	−7.94 ± 0.01	1.31 ± 0.00	Arg77, Arg109, Asp112, Leu113, Arg116, Ala172, Ser173, Gln174, Thr179, Ser182, Gln186, Arg187, Glu190, Gly191
**7** Paritaprevir	−9.87 ± 0.56	3.21 ± 1.90	Arg77, Pro78, Gly79, Ala80, Phe81, Thr102, Cys105, Arg109, Arg170, Gly171, Ala172, Ser173, Gln174, Thr179, Glu190, Phe194
**10** Conivaptan	−8.28 ± 0.01	1.12 ± 0.01	Tyr59, Ile62, Gln63, Arg109, Asp112, Leu113, Arg116, Leu117, Pro118, Thr179, Ser182, Arg187, Glu190, Gly191, Phe194
**11** Dihydroergotamine	−8.08 ± 0.01	2.56 ± 0.00	Arg77, Pro78, Gly79, Ala80, Arg109, Asp112, Leu113, Gly171, Ala172, Gln174, Pro178, Thr179, Ser182, Arg187, Glu190, Gly191, Arg193, Phe194
**12** Lumacaftor	−7.41 ± 0.20	1.34 ± 0.021	Arg77, Pro78, Gly79, Ala80, Phe81, Arg109, Gly171, Ala172, Gln174, Thr179, Ser182, Gln186, Arg187, Glu190, Arg193, Phe194
**13** Ivermectin	−10.48 ± 0.32	2.21 ± 0.35	Tyr59, Arg77, Pro78, Gly79, Ala80, Arg109, Asp112, Leu113, Arg116, Ala168, Gly169, Arg170, Gly171, Gln174, Pro178, Thr179, Ala181, Ser182, Asp183, Gln186, Arg187, Glu190, Gly191

**Table 3:** Docking scores of eight compounds against negative control proteins—Keratin (PDB Code: 6EC0), Ubiquitin (PDB Code: 8ST7), and Protein Kinase A (PDB Code: 1ATP)—obtained using MOE 2022.02, based on three independent docking runs, to validate binding specificity for Oncostatin M.

	Keratin	Ubiquitin	Protein Kinase A
Drug	Docking Score S	Docking Score S	Docking Score S
**1a** Ecamsule (1*S*,4*R*-1′*S*,4′*R*, C_2_-symmetric)	−5.56 ± 0.00	−5.31 ± 0.00	0.47 ± 0.00
**1b** Ecamsule (1*S*,4*R*-1′*R*,4′*S*, *meso*)	−5.93 ± 0.00	−5.39 ± 0.19	2.97 ± 0.44
**1c** Ecamsule (1*R*,4*S*-1′*R*,4′*S*, C_2_-symmetric)	−5.76 ± 0.00	−5.18 ± 0.00	2.75 ± 0.00
**7** Paritaprevir	−7.26 ± 0.21	−7.01 ± 0.56	28.24 ± 16.40
**10** Conivaptan	−6.09 ± 0.20	−5.90 ± 0.09	−8.79 ± 0.17
**11** Dihydroergotamine	−7.26 ± 0.23	−6.76 ± 0.26	7.03 ± 2.53
**12** Lumacaftor	−6.23 ± 0.00	−6.22 ± 0.00	−2.47 ± 0.00
**13** Ivermectin	−6.09 ± 0.00	−5.69 ± 0.00	−6.29 ± 0.00

**Table 4:** Properties of candidate drugs identified by virtual drug screening.

Drug	Fluorescence	Main Activity	Side Effects
**1a** Ecamsule	No	Sun protector	Dermatitis, itching
**2** Simeprevir	No	Hepatitis C	Respiratory problems, hepatotoxicity
**3** Telmisartan	No	Hypertension	Headache, nausea, diarrhea, muscle cramps, suspected carcinogenicity (rare)
**5** Sulfasalazine	Yellow	Inflammatory bowel diseases	Nausea, vomiting, diarrhea, rheumatism, abdominal pain
**6** Adapalene	Blue	Acne	Skin irritations
**7** Paritaprevir	No	Hepatitis C	Headache, nausea, angioedema (rare), liver failure (rare)
**8** Venetoclax	Yellow color	Cancer	Neutropenia, respiratory infections, nausea, vomiting, diarrhea
**9** Indacaterol	Yellow color	Chronic obstructive lung disease (COPD)	Headache, respiratory infections, muscle cramps, tachycardia (rare)
**10** Conivaptan	No	Hyponatremia	Headache, orthostatic hypotonia
**11** Dihydroergotamine	No	Migraine, vasoconstriction	Headache, nausea, vomiting, diarrhea, stroke (rare), peripheral ischemia (rare), allergies (rare)
**12** Lumacaftor	No	Cystic fibrosis	Headache, nausea, diarrhea, menstrual disorders
**13** Ivermectin	No	Parasite infections	Headache, nausea, diarrhea, fever, hypotonia

**Table 5:** Primer sequences of inflammatory marker genes for qRT-PCR.

Gene Name	Gene Symbol	Forward Primer	Reverse Primer
Interleukin 6	*IL6*	5′ CGGTCCAGTTGCCTTCTCC 3′	5′ ATTTGTGGTTGGGTCAGGGG 3′
Glyceraldehyde-3-phosphate dehydrogenase	*GAPDH*	5′ ATGAATGGGCAGCCGTTAGG 3′	5′ AGCATCACCCGGAGGAGAAA 3′
C-X-C Motif Chemokine	*CXCL11*	5′ AGGTGGGTGAAAGGACCAAA 3′	5′ GACTCCTTTGGGCAGTGGAA 3′
Tumor necrosis factor alpha	*TNFA*	5′ CAAGGACAGCAGAGGACCA 3′	5′ TGGCGTCTGAGGGTTGTTTT 3′

## Data Availability

The original contributions presented in the study are included in the article/Supplementary Material, further inquiries can be directed to the corresponding author.

## Supplementary Materials

The following supporting information can be downloaded at https://www.mdpi.com/article/10.3390/molecules30091897/s1, Table S1: Differentially expressed genes in ileum biopsies from patients with Crohn’s disease; Table S2: Differentially expressed genes in colon biopsies from patients with Crohn’s disease. Figure S1: ^1^H NMR spectrum of the commercially available sample of ecamsule (**1a**) used in this study, measured in methanol-d4. Figure S2: The raw data from three repetitions of the ecamsule (**1a**) MST experiment.
