# Supplementary material for: Drug Repurposing to Inhibit Oncostatin M in Crohn’s Disease
Source: Molecules. 2025 Apr 24;30(9):1897. doi: 10.3390/molecules30091897 (PMC12073679; doi:10.3390/molecules30091897)

Drug-Repurposing to Inhibit Oncostatin M in Crohn's Disease

Faranak Bahramimehr[1], Axel Guthart[1], Stefanie Kurz[1], Yuanping Hai[1], Mona Dawood[1,4], Rümeysa Yücer[1], Nasim Shahhamzehei[1], Ralf Weiskirchen[2], Wilfried Roth[3], Wolfgang Stremmel[5], Gerhard Bringmann[6], Thomas Efferth[1*]

* Corresponding author: Department of Pharmaceutical Biology, Institute of Pharmaceutical and Biomedical Sciences, Johannes Gutenberg University, Staudinger Weg 5, 55128 Mainz, Germany. Tel.: +49-6131-3925751; E-mail: efferth@uni-mainz.de

Figure S1: ^{1}H NMR spectrum of the commercially available sample of ecamsule (**1a**) used in this study, taken in methanol-d4.

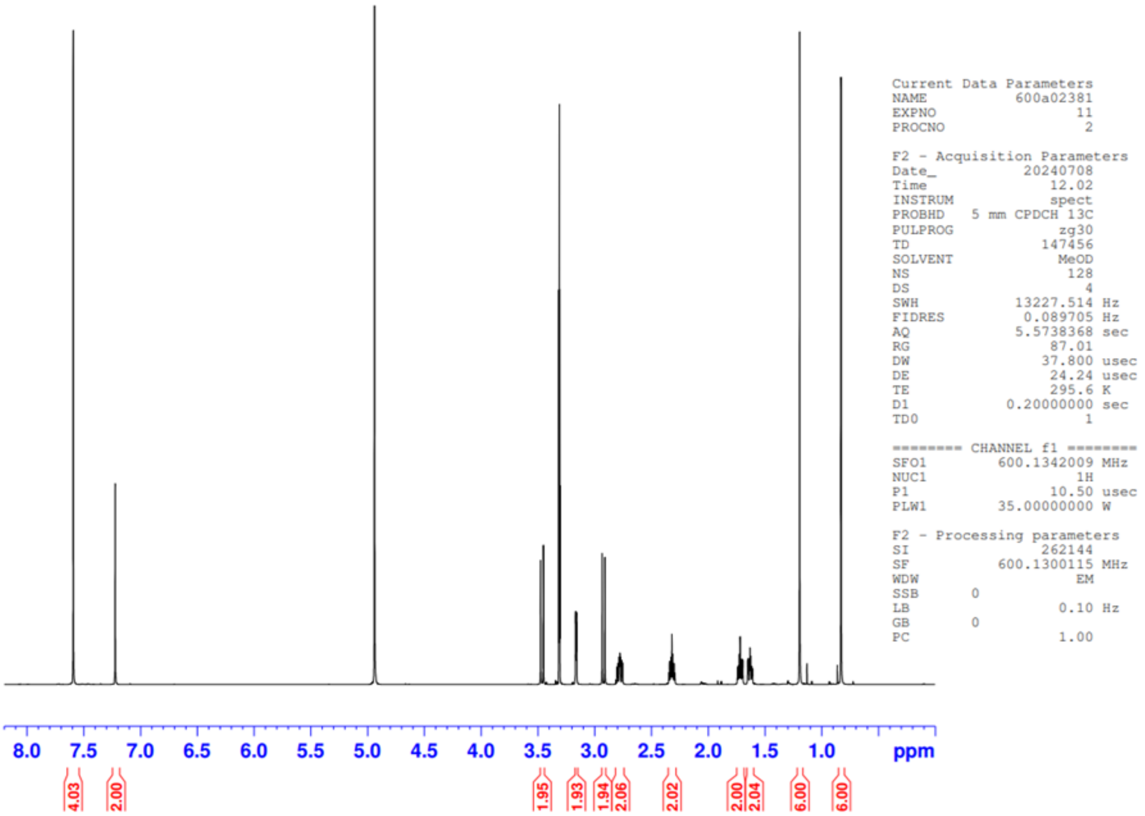

Supplement: Supplementary file 1 [file molecules-30-01897-s001.zip › Supplementary Figure S1.pdf]
