# Supplementary material for: Drug Repurposing to Inhibit Oncostatin M in Crohn’s Disease
Source: Molecules. 2025 Apr 24;30(9):1897. doi: 10.3390/molecules30091897 (PMC12073679; doi:10.3390/molecules30091897)
Supplement: Supplementary file 1 [file molecules-30-01897-s001.zip › Supplementary Figure S2 06-04-25.pdf]

Drug-Repurposing to Inhibit Oncostatin M in Crohn's Disease

Faranak Bahramimehr[1], Axel Guthart[1], Stefanie Kurz[1], Yuanping Hai[1], Mona Dawood[1,4], Rümeysa Yücer[1], Nasim Shahhamzehei[1], Ralf Weiskirchen[2], Wilfried Roth[3], Wolfgang Stremmel[5], Gerhard Bringmann[6], Thomas Efferth[1]*

* Corresponding author: Department of Pharmaceutical Biology, Institute of Pharmaceutical and Biomedical Sciences, Johannes Gutenberg University, Staudinger Weg 5, 55128 Mainz, Germany. Tel.: +49-6131-3925751; E-mail: efferth@uni-mainz.de

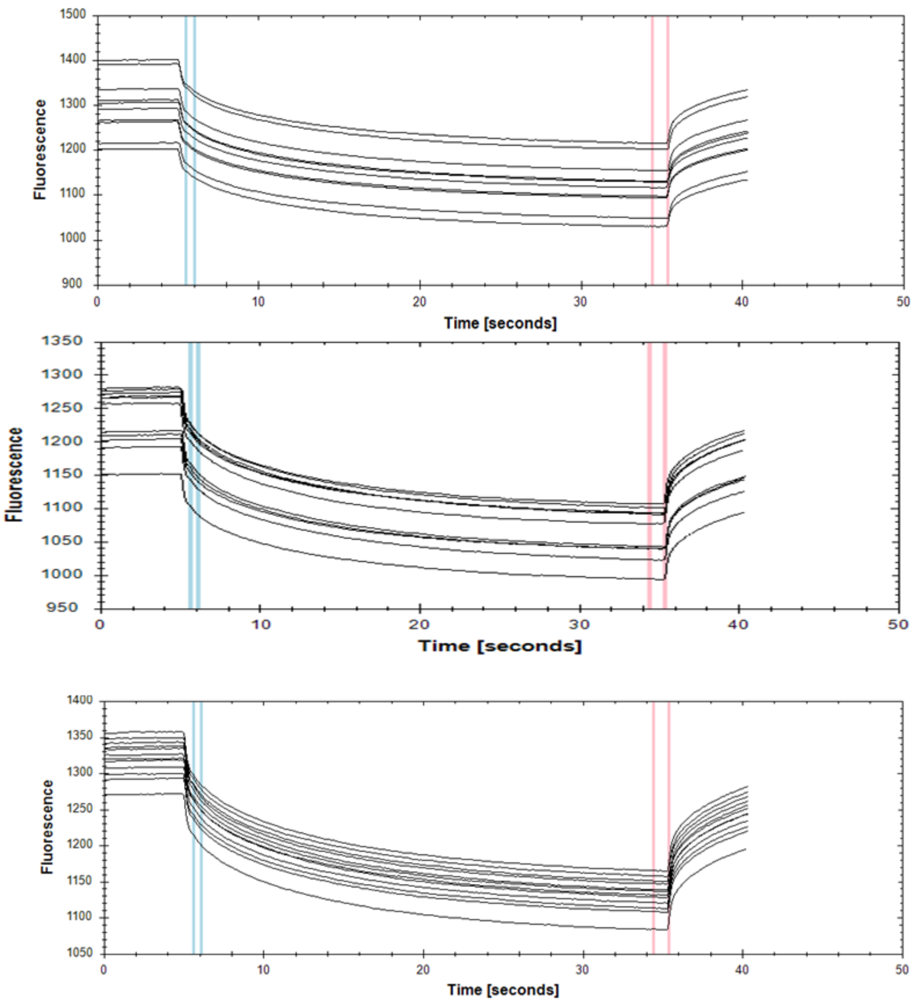

Figure S2: Raw data of 3 different microscale thermophoresis data with ecamsule **(1a)** with oncostatine M. The final protein concentration after labeling was 1000 nM in all repetitions. The titration was carried out using a wide concentration range of the compound (dilution steps 1:1). The DMSO concentration in all different ligand concentrations was stable (2.5%). The signals were measured using a Monolith NT.115 instrument (Nano Temper Technologies) with 20% light emitting diode (LED) power with MST power 10 for ecamsule. Fitting curves and K_d values of of 11.36 ± 2.83 μM
