# Supplementary material for: Drug Repurposing to Inhibit Oncostatin M in Crohn’s Disease
Source: Molecules. 2025 Apr 24;30(9):1897. doi: 10.3390/molecules30091897 (PMC12073679; doi:10.3390/molecules30091897)
Supplement: Supplementary file 1 [file molecules-30-01897-s001.zip › Supplementary Table S1 - Ileum conv.pdf]

Drug-Repurposing to Inhibit Oncostatin M in Crohn's Disease

Faranak Bahramimehr[1], Axel Guthart[1], Stefanie Kurz[1], Yuanping Hai[1], Mona Dawood[1,4], Rümeysa Yücer[1], Nasim Shahhamzehei[1], Ralf Weiskirchen[2], Wilfried Roth[3], Wolfgang Stremmel[5], Gerhard Bringmann[6], Thomas Efferth[1*]

* Corresponding author: Department of Pharmaceutical Biology, Institute of Pharmaceutical and Biomedical Sciences, Johannes Gutenberg University, Staudinger Weg 5, 55128 Mainz, Germany. Tel.: +49-6131-3925751; E-mail: efferth@uni-mainz.de

Table S1: Differentially expressed genes in ileum biopsies from patients with Crohn's disease. The positive fold-change values indicate upregulation compared to ileum biopsies from healthy individuals, the negative ones indicate downregulation.

Gene	Name	Fold change
**Upregulated genes:**		
FOLH1	Glutamate carboxypeptidase	5.28
MUC6	Mucin-6	4.19
CCL19	Chemokine C-C motif ligand 19	4.11
UBD	Ubiquitin D	3.94
MMP1	Interstitial collagenase	3.82
DUOXA2	Dual oxidase maturation factor 2	3.74
DUOX2	Dual oxidase 2	3.71
CXCL13	Chemokine (C-X-C motif) ligand 13	3.69
C3	Complement component 3	3.62
MMP12	Macrophage metalloelastase	3.44
IL1B	Interleukin-1 β	3.35
TCL1A	T-cell leukemia/lymphoma protein 1A	3.29
CD19	B-lymphocyte antigen CD19	3.27
HCAR3	Hydroxycarboxylic acid receptor 3	3.25
IGHG1	Ig γ-1 chain C region	3.21
S100A8	Protein S100-A8	3.20
CCR7	C-C chemokine receptor type 7	3.15
NPC1L1	Niemann-Pick C1-like protein 1	3.08
CR2	Complement receptor type 2	3.05
MMP9	Matrix metalloproteinase-9	2.93
CXCL9	C-X-C motif chemokine 9	2.91
IGHM	Ig μ chain C region	2.86
ADH4	Alcohol dehydrogenase	2.75
NOS2	Nitric oxide synthase, inducible	2.70
IL4I1	L-amino-acid oxidase	2.62
LAMP3	Lysosome-associated membrane glycoprotein	2.62
FCRL3	Fc receptor-like protein 3	2.61
PTGDS	Prostaglandin-H2 D-isomerase	2.60
BANK1	B-cell scaffold protein with ankyrin repeats	2.58
CCL17	C-C motif chemokine 17	2.58
CD83	CD83 antigen	2.58

CXCR4	C-X-C chemokine receptor type 4	2.58
FCGR3A	Low afinity immunoglobulin γ Fc region receptor III-A	2.53
FCRL5	Fc receptor-like protein 5	2.46
MUC1	Mucin-1	2.39
FPR1	fMet-Leu-Phe receptor	2.38
LTF	Lactotransferrin	2.35
MS4A1	B-lymphocyte antigen CD20	2.35
CXCR5	C-X-C chemokine receptor type 5	2.34
SAA2	Serum amyloid A2	2.32
LCT	Lactase	2.31
POU2AF1	POU domain class 2-associating factor 1	2.30
CCL21	C-C motif chemokine 21	2.29
SELL	L-selectin	2.28
HAPLN3	Hyaluronan and proteoglycan link protein 3	2.27
LTB	Lymphotoxin β (TNF superfamily, member 3)	2.24
GIP	Gastric inhibitory polypeptide	2.23
CTSE	Cathepsin E	2.21
CXCL1	Growth-regulated alpha protein	2.17
CA1	Carbonic anhydrase 1	2.16
AIM2	Interferon-inducible protein AIM2	2.13
TFF2	Trefoil factor 2	2.12
ADAMTS4	A disintegrin and metalloproteinase with thrombospondin motifs 4	2.11
FSCN1	Fascin	2.10
TNFSF11	Tumor necrosis factor ligand superfamily member 11	2.09
BLK	Tyrosine-protein kinase Blk	2.08
ITGAX	Integrin α-X 2.07	
OSM	Oncostatin-M	2.06
FCRLA	Fc receptor-like A	2.03
LPL	Lipoprotein lipase	2.03
IL1RN	Interleukin-1 receptor antagonist protein	2.01
CCL23	C-C motif chemokine 23	2.01
**Downregulated genes:**		
SRD5A3	Probable polyprenol reductase	-2.10
KIF3C	Kinesin-like protein KIF3C	-2.14
GPR144	Probable G-protein coupled receptor 144	-2.23
DNAH12	Dynein heavy chain 12, axonemal	-2.32
CRABP1	Cellular retinoic acid-binding protein 1	-2.34
CCL20	C-C motif chemokine 20	-2.44
C10orf128	Putative uncharacterized protein C10orf128	-2.52
FOXD1	Forkhead box protein D1	-2.80
XPO5	Exportin-5	-2.83
TNK2	Activated CDC42 kinase 1	-3.32
