# Supplementary material for: Drug Repurposing to Inhibit Oncostatin M in Crohn’s Disease
Source: Molecules. 2025 Apr 24;30(9):1897. doi: 10.3390/molecules30091897 (PMC12073679; doi:10.3390/molecules30091897)
Supplement: Supplementary file 1 [file molecules-30-01897-s001.zip › Supplementary Table S2 - Colon.pdf]

Drug-Repurposing to Inhibit Oncostatin M in Crohn's Disease

Faranak Bahramimehr[1], Axel Guthart[1], Stefanie Kurz[1], Yuanping Hai[1], Mona Dawood[1,4], Rümeysa Yücer[1], Nasim Shahhamzehei[1], Ralf Weiskirchen[2], Wilfried Roth[3], Wolfgang Stremmel[5], Gerhard Bringmann[6], Thomas Efferth[1*]

* Corresponding author: Department of Pharmaceutical Biology, Institute of Pharmaceutical and Biomedical Sciences, Johannes Gutenberg University, Staudinger Weg 5, 55128 Mainz, Germany. Tel.: +49-6131-3925751; E-mail: efferth@uni-mainz.de

Table S2: Differentially expressed genes in colon biopsies from patients with Crohn's disease. The positive fold-change values indicate upregulation compared to ileum biopsies from healthy individuals, the negative ones indicate downregulation.

Gene	Name	Fold change
**Upregulated genes:**		
REG1B	Lithostathine-1-β	4.04
S100A8	Protein S100-A8	3.76
HCAR3	Hydroxycarboxylic acid receptor 3	3.51
TCL1A	T-cell leukemia/lymphoma protein 1A	3.37
OXCT2	Succinyl-CoA:3-ketoacid-coenzyme A transferase 2, mitochondrial	3.27
C10orf122	Putative uncharacterized protein C10orf122	3.16
FAM65B	Protein FAM65B	3.16
ST5	Suppression of tumorigenicity 5 protein	2.98
PSCA	Prostate stem cell antigen	2.81
HS3ST3B1	Heparan sulfate glucosamine 3-O-sulfotransferase 3B1	2.67
PPP1R2	Protein phosphatase inhibitor 2	2.53
NRBP2	Nuclear receptor-binding protein 2	2.45
TM2D3	TM2 domain-containing protein 3	2.08
CYP4F11	Cytochrome P450 4F11	2.05
OSM	Oncostatin-M	2.04
KIRREL3	Kirre-like nephrin family adhesion molecule 3	1.98
PDS5A	Sister chromatid cohesion protein PDS5 homolog A	1.98
PLA2G2A	Phospholipase A2, membrane associated	1.90
CLEC12A	C-type lectin domain family 12 member A	1.88
MDGA1	MAM domain-containing glycosyl-phosphatidylinositol anchor protein 1	1.84
*IGHG*1	Ig γ-1 chain C region	1.82
CXCL5	C-X-C motif chemokine 5	1.81
CYP2A6	Cytochrome P450 2A6	1.81
IL1B	Interleukin-1 β	1.80
ZNF138	Zinc finger protein 138	1.80

Downregulated genes:

CD86	T-lymphocyte activation antigen CD86	-1.92
FOSL1	Fos-related antigen 1	-2.06
MT1E	Metallothionein-1E	-2.08
GAL	Galanin prepropeptide	-2.30
SLC37A2	Sugar phosphate exchanger 2	-2.54
PLCXD1	PI-PLC X domain-containing protein 1	-2.59
KIT	Mast/stem cell growth factor receptor Kit	-2.61
IGSF1	Immunoglobulin superfamily member 1	-3.81
